# Deep learning-based reduced order models in cardiac electrophysiology

**DOI:** 10.1371/journal.pone.0239416

**Published:** 2020-10-01

**Authors:** Stefania Fresca, Andrea Manzoni, Luca Dedè, Alfio Quarteroni

**Affiliations:** 1 MOX - Dipartimento di Matematica, Politecnico di Milano, Milano, Italy; 2 Mathematics Institute, École Polytechnique Fédérale de Lausanne, Lausanne, Switzerland; Queensland University of Technology, AUSTRALIA

## Abstract

Predicting the electrical behavior of the heart, from the cellular scale to the tissue level, relies on the numerical approximation of coupled nonlinear dynamical systems. These systems describe the cardiac action potential, that is the polarization/depolarization cycle occurring at every heart beat that models the time evolution of the electrical potential across the cell membrane, as well as a set of ionic variables. Multiple solutions of these systems, corresponding to different model inputs, are required to evaluate outputs of clinical interest, such as activation maps and action potential duration. More importantly, these models feature coherent structures that propagate over time, such as wavefronts. These systems can hardly be reduced to lower dimensional problems by conventional reduced order models (ROMs) such as, e.g., the reduced basis method. This is primarily due to the low regularity of the solution manifold (with respect to the problem parameters), as well as to the nonlinear nature of the input-output maps that we intend to reconstruct numerically. To overcome this difficulty, in this paper we propose a new, nonlinear approach relying on deep learning (DL) algorithms—such as deep feedforward neural networks and convolutional autoencoders—to obtain accurate and efficient ROMs, whose dimensionality matches the number of system parameters. We show that the proposed DL-ROM framework can efficiently provide solutions to parametrized electrophysiology problems, thus enabling multi-scenario analysis in pathological cases. We investigate four challenging test cases in cardiac electrophysiology, thus demonstrating that DL-ROM outperforms classical projection-based ROMs.

## Introduction

The electrical activation of the heart, which drives its contraction, is the result of two processes: at the microscopic scale, the generation of ionic currents through the cellular membrane producing a local action potential; and at the macroscopic scale, the propagation of the action potential from cell to cell in the form of a transmembrane potential [1–3]. This latter process can be described by means of partial differential equations (PDEs), suitably coupled with systems of ordinary differential equations (ODEs) modeling the ionic currents in the cells.

Solving this system using a high-fidelity, full order model (FOM) such as, e.g., the finite element (FE) method, is computationally demanding. Indeed, the propagation of the electrical signal is characterized by the fast dynamics of very steep fronts, thus requiring very fine space and time discretizations [3–5]; see also, e.g., [6, 7] for higher-order and/or more robust numerical methods, [8] for time and space adaptivity, and [9] regarding the use of GPU computing in this context. Using a FOM may quickly become unaffordable if such a coupled system must be solved for several values of parameters representing either functional or geometric data such as, e.g., material properties, initial and boundary conditions, or the shape of the domain. Multi-query analysis is relevant in a variety of situations: when analyzing multiple scenarios, when dealing with sensitivity analysis and uncertainty quantification (UQ) problems in order to account for inter-subject variability [10–13], for parameter estimation or data assimilation, in which some unknown (or unaccessible) quantities characterizing the mathematical model must be inferred from a set of measurements [14–18]. In all these cases, to achieve computational efficiency, multi-query analysis in cardiac electrophysiology must rely on suitable surrogate models see, e.g., [19] for a recent review on the topic. Among surrogate models, several options are available, such as *(i)* emulators, obtained, e.g., via Polynomial Chaos Expansions or Gaussian process regression [20–22], aiming at the approximation of the input-output mapping by fitting a set of training data; *(ii)* lower-fidelity models, introducing suitable modeling simplifications—such as, for instance, the Eikonal model in this context [23]; and *(iii)* reduced order models (ROMs) obtained through a projection process on the equations governing the FOM to reduce the state-space dimensionality. Although typically more intrusive to implement, ROMs often yield more accurate approximations than data fitting and usually generate more significant computational gains than lower-fidelity models.

Conventional projection-based ROMs built, e.g., through the reduced basis (RB) method [24], yields inefficient ROMs when dealing with nonlinear time-dependent parametrized PDE-ODE system as the one arising from cardiac electrophysiology. The three major computational bottlenecks shown by such kind of ROMs for cardiac electrophysiology are:

- the linear superimposition of modes, on which they are based, would cause the dimension of the ROM to be excessively large to guarantee an acceptable accuracy;
- evaluating the ROM requires the solution of a dynamical system, which might be unstable unless the size of time step Δ*t* is very small;
- the ROM must also account for the dynamics of the gating variables, even when aiming at computing just the electrical potential. This fact entails an extremely intrusive and costly hyper-reduction stage to reduce the solution of the ODE system to a few, selected mesh nodes [25].

To overcome the limitations of projection-based ROMs, we propose a new, non-intrusive ROM technique based on deep learning (DL) algorithms, which we refer to as DL-ROM. Combining in a suitable way a convolutional autoencoder (AE) and a deep feedforward neural network (DFNN), the DL-ROM technique enables the construction of an efficient ROM, whose dimension is as close as possible to the number of parameters upon which the solution of the differential problem depends. A preliminary numerical assessment of our DL-ROM technique has already been presented in [26], albeit on simpler—yet challenging—test cases.

The proposed DL-ROM technique is a combination of a data-driven with a physics based model approach. Indeed, it exploits snapshots taken from a set of FOM solutions (for selected parameter values and time instances) and deep neural network architectures to learn, in a non-intrusive way, both *(i)* the nonlinear trial manifold where the ROM solution is sought, and *(ii)* the nonlinear reduced dynamics. In a linear ROM built, e.g., through proper orthogonal decomposition (POD), the former quantity is nothing but a set of basis functions, while the latter task corresponds to the projection stage in the subspace spanned by these basis functions. Here, our goal is to show that DL-ROM can be effectively used to handle parametrized problems in cardiac electrophysiology, accounting for both physiological and pathological conditions, in order to provide fast and accurate solutions. The proposed DL-ROM is computationally efficient during the testing stage, that is for any new scenario unseen during the training stage. This is particularly useful in view of the evaluation of patient-specific features to enable the integration of computational methods in current clinical platforms.

DL techniques for parametrized PDEs have previously been proposed in other contexts. In [27–30] feedforward neural networks have been employed to model the reduced dynamics in a less intrusive way, that is, avoiding the costs entailed by projection-based ROMs, but still relying on a linear trial manifold built, e.g., through POD. In [31–33] the construction of ROMs for nonlinear, time-dependent problems has been replaced by the evaluation of regression models based on artificial neural networks (ANNs). In [34, 35] the reduced trial manifold where the approximation is sought has been modeled through ANNs thus avoiding the linear superimposition of POD modes, on a minimum residual formulation to derive the ROM [35], or without considering an explicit parameter dependence in the differential problem that is considered [34]. In all these works, coupled problems have never been considered. Moreover, very often DL techniques have been exploited to address problems which require only a moderate dimension of projection-based ROMs. We demonstrate that our DL-ROM provides accurate results by constructing ROMs with extremely low-dimension in prototypical test cases. These tests exhibit all the relevant physical features which make the numerical approximation of parametrized problems in cardiac electrophysiology a challenging task.

## Materials and methods

### Cardiac electrophysiology

Muscle contraction and relaxation drive the pump function of the heart. In particular, tissue contraction is triggered by electrical signals self-generated in the heart and propagated through the myocardium thanks to the excitability of the cardiac cells, the cardiomyocites [3, 36]. When suitably stimulated, cardiomyocites produce a variation of the potential across the cellular membrane, called *transmembrane potential*. Its evolution in time is usually referred to as *action potential*, involving a polarization and a depolarization in the early stage of every heart beat. The action potential is generated by several ion channels (e.g., calcium, sodium, potassium) that open and close, and by the resulting ionic currents crossing the membrane. For instance, coupling the so-called monodomain model for the transmembrane potential *u* = *u*(***x***, *t*) with a phenomenological model for the ionic currents—involving a single gating variable *w* = *w*(***x***, *t*)—in a domain Ω representing, e.g., a portion of the myocardium, results in the following nonlinear time-dependent system

$$\left\{ \begin{array}{c} \begin{array}{cc} \frac{\partial u}{\partial t}-\text{div}\left(D\nabla u\right)+I_{ion}\left(u,w\right)=I_{app}\left(x,t\right)\; & (x,t)\in \Omega \times (0,T),\\ \frac{\partial w}{\partial t}+g\left(u,w\right)=0\; & (x,t)\in \Omega \times (0,T),\\ \nabla u\cdot n=0\; & (x,t)\in \partial \Omega \times (0,T),\\ u(x,0)=0,\;w(x,0)=0\; & x\in \Omega . \end{array} \end{array}\right.$$ (1)

Here *t* denotes a rescaled time Dimensional times and potential [37] are given by $\tilde{t}\left[ms\right]=12.9t$ and $\tilde{u}\left[mV\right]=100u-80$. The transmembrane potential ranges from the resting state of −80 mV to the excited state of + 20 mV., **n** denotes the outward directed unit vector normal to the boundary ∂Ω of Ω, whereas *I*_*app*_ is an applied current representing, e.g., the initial activation of the tissue. The nonlinear diffusion-reaction equation for *u* is two-ways coupled with the ODE system, which must be in principle solved at any point **x** ∈ Ω; indeed, the reaction term *I*_*ion*_ and the function *g* depend on both *u* and *w*. The most common choices for the two functions *I*_*ion*_ and *g* in order to efficiently reproduce the action-potential are, e.g., the FitzHugh-Nagumo [38, 39], the Aliev-Panfilov [37, 40] or the Mitchell and Schaeffer models [41]. The diffusivity tensor **D** usually depends on the fibers-sheet structure of the tissue, affecting directional conduction velocities and directions. In particular, by assuming an axisymmetric distribution of the fibers, the conductivity tensor takes the form

$$\begin{array}{c} D\left(x\right)=\sigma_{t}I+\left(\sigma_{l}-\sigma_{t}\right)f_{0}⊗f_{0}, \end{array}$$ (2)

where *σ*_*l*_ and *σ*_*t*_ are the conductivities in the fibers and the transversal directions.

When a simple phenomenological ionic model is considered, such as the FitzHugh-Nagumo or the Aliev-Panfilov (A-P) model, the ionic current takes the form of a cubic nonlinear function of *u* and a single (dimensionless) gating variable plays the role of a recovery function, allowing to model refractoriness of cells. In this paper, we focus on the Aliev-Panfilov model, which consists in taking

$$\begin{array}{c} \begin{array}{cc} & I_{ion}\left(u,w\right)=Ku\left(u-a\right)\left(u-1\right)+uw,\\ & g\left(u,w\right)=(\epsilon_{0}+\frac{c_{1}w}{c_{2}+u})\left(-w-Ku\left(u-b-1\right)\right). \end{array} \end{array}$$ (3)

The parameters *K*, *a*, *b*, *ε*_0_, *c*_1_, *c*_2_ are related to the cell. Here *a* represents an oscillation threshold, whereas the weighting factor $\varepsilon_{0}+\frac{c_{1}w}{c_{2}+u}$ was introduced in [37] to tune the restitution curve to experimental observations by adjusting the parameters *c*_1_ and *c*_2_; see, e.g., [1–3, 42] for a detailed review. In the remaining part of the paper, we denote by $\mu \in P\subset R^{n_{\mu }}$ a parameter vector listing all the *n_**μ**_* input parameters characterizing physical (and, possibly, geometrical) properties we might be interested to vary; P is a subset of $R^{n_{\mu }}$, denoting the parameter space. Relevant physical situations are those in which input parameters affect the diffusivity matrix **D** (through the conduction velocities) and the applied current *I*_*app*_; previous analyses focused instead on the gating variable dynamics (through *g*) and the ionic current *I*_*ion*_, see [25].

### Projection-based ROMs

From an algebraic standpoint, the spatial discretization of system (1) through the Galerkin-finite element (FE) approximation [43] yields the following nonlinear dynamical system for **u** = **u**(*t*, ***μ***), **w** = **w**(*t*, ***μ***), representing our full order model (FOM):

$$\left\{ \begin{array}{c} \begin{array}{cc} M\left(\mu \right)\frac{\partial u}{\partial t}+A\left(\mu \right)u+I_{ion}\left(t,u,w;\mu \right)=I_{app}\left(t;\mu \right), & \;\;t\in (0,T),\\ \frac{\partial w}{\partial t}\left(t;\mu \right)=g\left(t,u,w;\mu \right), & \;\;t\in (0,T),\\ u(0)=0,\;w(0)=0. \end{array} \end{array}\right.$$ (4)

Here $A\left(\mu \right)\in R^{N\times N}$ is a matrix arising from the diffusion operator (thus including the conductivity tensor **D**(***μ***) = **D**(**x**; ***μ***), which can vary within the myocardium due to fiber orientation and conditions, such as the possible presence of ischemic regions); $M\left(\mu \right)\in R^{N\times N}$ is the mass matrix; $I_{ion},g\in R^{N}$ are vectors arising from the nonlinear terms; finally, $I_{app}\in R^{N}$ is a vector collecting the applied currents. The dimension *N* is related to the dimension of the FE space and, ultimately, depends on the size *h* > 0 of the computational mesh used to discretize the domain Ω. Note that the system of ODEs arises from the collocation of the ODE in (1) at the nodes used for the numerical integration. A detailed derivation of the FOM (4) is reported in the S1 Appendix.

The intrinsic dimension of the solution manifold

$$\begin{array}{c} S=\left\{u\left(t;\mu \right)\;|\;t\in \left[0,T\right)\;\text{and}\;\mu \in P\subset R^{n_{\mu }}\right\}\subset R^{N}, \end{array}$$ (5)

obtained by solving (4) when (*t*; ***μ***) varies in [0,T)×P, is usually much smaller than *N* and, under suitable conditions, is at most *n*_***μ***_ + 1 ≪ *N*, where *n*_***μ***_ is the number of parameters—in this respect, the time independent variable plays the role of a parameter. For this reason, ROMs attempt at approximating S by introducing a suitable trial manifold of lower dimension. The most popular approach is *proper orthogonal decomposition* (POD), which exploits a *linear trial manifold* built through the singular value decomposition of a matrix $S\in R^{N\times N_{s}}$ collecting a set of FOM snapshots

$$\begin{array}{c} S=[u\left(t^{1};\mu_{1}\right)\;|\;\ldots \;|\;u\left(t^{N_{t}};\mu_{1}\right)\;|\;\ldots \;|\;u\left(t^{1};\mu_{N_{train}}\right)\;\left|\;\ldots \;\right|\;u\left(t^{N_{t}};\mu_{N_{train}}\right)]; \end{array}$$

this is a set of solutions obtained for *N*_*train*_ selected input parameters at (a subset, possibly, of) the time instants ${\left\{t^{k}\right\}}_{k=1}^{N_{t}}$ in which (0, *T*) is partitioned for the sake of time discretization. The most common choice is to set *t*^*k*^ = *k*Δ*t* where Δ*t* = *T*/(*N*_*t*_ − 1).

When using a projection-based ROM, the approximation of **u**(*t*; ***μ***) is sought as a linear superimposition of modes, under the form

$$\begin{array}{c} u\left(t;\mu \right)\approx Vu_{n}\left(t;\mu \right), \end{array}$$ (6)

thus yielding a linear ROM, in which the columns of the matrix $V=\left[\zeta_{1},\ldots ,\zeta_{n}\right]\in R^{N\times n}$ form an orthonormal basis of a space *V*_*n*_, an *n*-dimensional subspace of $R^{N}$. In the case of POD, *V*_*n*_ provides the best *n*-rank approximation of ***S*** in the Frobenius norm, that is, **ζ**_1_, …, **ζ**_*n*_ are the first *n* (left) singular vectors of ***S*** corresponding to the *n* largest singular values *σ*_1_, …, *σ*_*n*_ of ***S***, such that the projection error is smaller than a desired tolerance *ε*_*POD*_. To meet this requirement, it is sufficient to choose *n* as the smallest integer such that

$$\begin{array}{c} \frac{\sum_{i=1}^{N}\sigma_{i}^{2}}{\sum_{i=1}^{N_{s}}\sigma_{i}^{2}}>1-\varepsilon_{POD}^{2}, \end{array}$$

i.e., the energy retained by the last *N*_*s*_ − *n* POD modes is equal or smaller than $\varepsilon_{POD}^{2}$.

The approximation of **w** is given instead by its restriction

$$\begin{array}{c} w\left(t;\mu \right)\approx Pw_{m}\left(t;\mu \right), \end{array}$$

to a (possibly, small) subset of *m* degrees of freedom, where *m* ≪ *n*, at which the nonlinear term **I**_*ion*_ is interpolated exploiting a problem-dependent basis, spanned by the columns of a matrix $\Phi \in R^{N\times m}$, which is built according to a suitable *hyper-reduction* strategy; see, e.g., [25] for further details. Here $P=\left[e_{1},\ldots ,e_{m}\right]\in R^{N\times m}$ denotes a matrix formed by the columns of the *N* × *N* identity matrix corresponding to the *m* selected degrees of freedom.

A Galerkin-POD ROM for system (1) is then obtained by *(i)* first, substituting (Eq 6) into Eq 4 and projecting it onto *V*_*n*_; then, *(ii)* solving the system of ODEs at *m* selected degrees of freedom, thus yielding the following nonlinear dynamical system for **u**_*n*_ = **u**_*n*_(*t*, ***μ***) and the selected components **P**^*T*^ **w** = **P**^*T*^ **w**(*t*; ***μ***) of ***w***:

$$\left\{ \begin{array}{c} \begin{array}{cc} V^{T}M\left(\mu \right)V\frac{\partial u_{n}}{\partial t}+V^{T}A\left(\mu \right)V^{T}u_{n}\\ \;+V^{T}\Phi {\left(P^{T}\Phi \right)}^{-1}I_{ion}\left(t,P^{T}Vu_{n},P^{T}w;\mu \right)-V^{T}I_{app}\left(t;\mu \right)=0, & t\in (0,T),\\ P^{T}\frac{\partial w}{\partial t}+g\left(t,P^{T}Vu_{n},P^{T}w;\mu \right)=0, & t\in (0,T),\\ u_{n}\left(0\right)=0,\;P^{T}w\left(0\right)=0. \end{array} \end{array}\right.$$ (7)

This strategy is the essence of the reduced basis (RB) method for nonlinear time-dependent parametrized PDEs. However, using (7) as an approximation to (4) is known to suffer from several problems. First of all, an extensive hyper-reduction stage (exploiting, e.g., the discrete empirical interpolation method (DEIM)) must be performed in order to be able to evaluate any ***μ***- or **u**-dependent quantities appearing in (7), that is, without relying on *N*-dimensional arrays. Moreover, whenever the solution of the differential problem features coherent structures that propagate over time, such as steep wavefronts, the dimension *n* of the projection-based ROM (7) might easily become very large, due to the basic linearity assumption, by which the solution is given by a linear superimposition of POD modes, thus severely degrading the computational efficiency of the ROM. A possible way to overcome this bottleneck is to rely on local reduced bases, built through POD after the set of snapshots has been split into *N*_*c*_ > 1 clusters, according to suitable clustering (or unsupervised learning) algorithms [25].

### Deep learning-based reduced order modeling (DL-ROM)

To overcome the limitations of linear ROMs, we consider a new, nonlinear ROM technique based on deep learning models. First introduced in [26] and assessed on one-dimensional benchmark problems, the DL-ROM technique aims at learning both the nonlinear trial manifold (corresponding to the matrix **V** in the case of a linear ROM) in which we seek the solution to the parametrized system (1) and the nonlinear reduced dynamics (corresponding to the projection stage in a linear ROM). This method is not intrusive; it relies on DL algorithms trained on a set of FOM solutions obtained for different parameter values.

We denote by *N*_*train*_ and *N*_*test*_ the number of training and testing parameter instances, respectively; the ROM dimension is again denoted by *n* ≪ *N*. In order to describe the system dynamics on a suitable reduced nonlinear trial manifold (a task which we refer to as *reduced dynamics learning*), the intrinsic coordinates of the ROM approximation are defined as

$$\begin{array}{c} u_{n}\left(t;\mu ,\theta_{DF}\right)=\phi_{n}^{DF}\left(t;\mu ,\theta_{DF}\right), \end{array}$$ (8)

where $\phi_{n}^{DF}\left(\cdot ;\cdot ,\theta_{DF}\right):R^{\left(n_{\mu }+1\right)}\to R^{n}$ is a DFNN, consisting in the subsequent composition of a nonlinear activation function, applied to a linear transformation of the input, multiple times [44]. Here ***θ***_*DF*_ denotes the vector of parameters of the DFNN, collecting all the corresponding weights and biases of each layer of the DFNN.

Regarding instead the description of the reduced nonlinear trial manifold, approximating the solution one, $\tilde{S}\approx S$ (a task which we refer to as *reduced trial manifold learning*) we employ the *decoder function of a convolutional autoencoder* The AE is a particular type of neural network aiming at learning the identity function

$$\begin{array}{c} f^{AE}\left(\cdot ;\theta_{E},\theta_{D}\right):x\mapsto \tilde{x}\;\text{with}\;\tilde{x}≃x. \end{array}$$ (9)

It is composed by two main parts:

- the *encoder* function $f_{n}^{E}\left(\cdot ;\theta_{E}\right):x\mapsto {\tilde{x}}_{n}=f_{n}^{E}\left(x;\theta_{E}\right)$, where $f_{n}^{E}\left(\cdot ;\theta_{E}\right):R^{N}\to R^{n}$ and *n* ≪ *N*, mapping the high-dimensional input **x** onto a low-dimensional code ${\tilde{x}}_{n}$;
- the *decoder* function $f^{D}\left(\cdot ;\theta_{D}\right):{\tilde{x}}_{n}\mapsto \tilde{x}=f^{D}\left({\tilde{x}}_{n};\theta_{D}\right)$, where $f^{D}\left(\cdot ;\theta_{D}\right):R^{n}\to R^{N}$, mapping the low-dimensional code ${\tilde{x}}_{n}$ to an approximation of the original high-dimensional input $\tilde{x}$.

(AE) [45, 46]. More precisely, $\tilde{S}$ takes the form

$$\begin{array}{c} \tilde{S}=\left\{f^{D}\left(u_{n}\left(t;\mu ,\theta_{DF}\right);\theta_{D}\right)\;|\;u_{n}\left(t;\mu ,\theta_{DF}\right)\in R^{n},\;t\in \left[0,T\right)\;\text{and}\;\mu \in P\subset R^{n_{\mu }}\right\} \end{array}$$ (10)

where $f^{D}\left(\cdot ;\theta_{D}\right):R^{n}\to R^{N}$ consists in the decoder function of a convolutional AE. This latter results from the composition of several layers (some of which are convolutional), depending upon a vector ***θ***_*D*_ collecting all the corresponding weights and biases.

As a matter of fact, the approximation $\tilde{u}\left(t;\mu \right)\approx u\left(t;\mu \right)$ provided by the DL-ROM technique is defined as

$$\begin{array}{c} \tilde{u}\left(t;\mu ,\theta_{DF},\theta_{D}\right)=f^{D}\left(\phi_{n}^{DF}\left(t;\mu ,\theta_{DF}\right);\theta_{D}\right). \end{array}$$ (11)

The encoder function of the convolutional AE can then be exploited to map the FOM solution associated to (*t*, ***μ***) onto a low-dimensional representation

$$\begin{array}{c} {\tilde{u}}_{n}\left(t;\mu ,\theta_{E}\right)=f_{n}^{E}\left(u\left(t;\mu \right);\theta_{E}\right); \end{array}$$ (12)

$f_{n}^{E}\left(\cdot ;\theta_{E}\right):R^{N}\to R^{n}$ denotes the encoder function, depending upon a vector ***θ***_*E*_ of parameters.

Computing the DL-ROM approximation of **u**(*t*; ***μ**_text_*), for any possible *t* ∈ (0, *T*) and $\mu_{test}\in P$, corresponds to the testing stage of a DFNN and of the decoder function of a convolutional AE; this does not require the evaluation of the encoder function. We remark that our DL-ROM strategy overcomes the three major computational bottlenecks implied by the use of projection-based ROMs, since:

- the dimension of the DL-ROM can be kept extremely small;
- the time resolution required by the DL-ROM can be chosen to be larger than the one required by the numerical solution of dynamical systems in cardiac electrophysiology;
- the DL-ROM can be queried at any desired time instant, without requiring the solution of a dynamical system until that time;
- the DL-ROM does not require to account for the dynamics of the gating variables, thus avoiding any hyper-reduction stage. This advantage, already visible when employing a single gating variable as in our case, might become even more effective when dealing with more realistic ionic models, when dozens of additional variables in the system of ODEs must be accounted for [3].

The training stage consists in solving the following optimization problem, in the variable ***θ*** = (***θ***_*E*_, ***θ***_*DF*_, ***θ***_*D*_), after the snapshot matrix ***S*** has been formed:

$$\begin{array}{c} \min_{\theta }J\left(\theta \right)=\min_{\theta }\frac{1}{N_{s}}\sum_{i=1}^{N_{train}}\sum_{k=1}^{N_{t}}L\left(t^{k},\mu_{i};\theta \right), \end{array}$$ (13)

where *N*_*s*_ = *N*_*train*_ *N*_*t*_ and

$$\begin{array}{c} \begin{array}{cc} L(t^{k},\mu_{i};\theta ) & =\frac{\omega_{h}}{2}{\left\|u\left(t^{k};\mu_{i}\right)-\tilde{u}\left(t^{k};\mu_{i},\theta_{DF},\theta_{D}\right)\right\|}^{2}\\ & +\frac{1-\omega_{h}}{2}{\left\|{\tilde{u}}_{n}\left(t^{k};\mu_{i},\theta_{E}\right)-u_{n}\left(t^{k};\mu_{i},\theta_{DF}\right)\right\|}^{2}, \end{array} \end{array}$$ (14)

with *ω*_*h*_ ∈ [0, 1]. The *per-example* loss function (14) combines the reconstruction error (that is, the error between the FOM solution and the DL-ROM approximation) and the error between the intrinsic coordinates and the output of the encoder.

**Fig 1:**
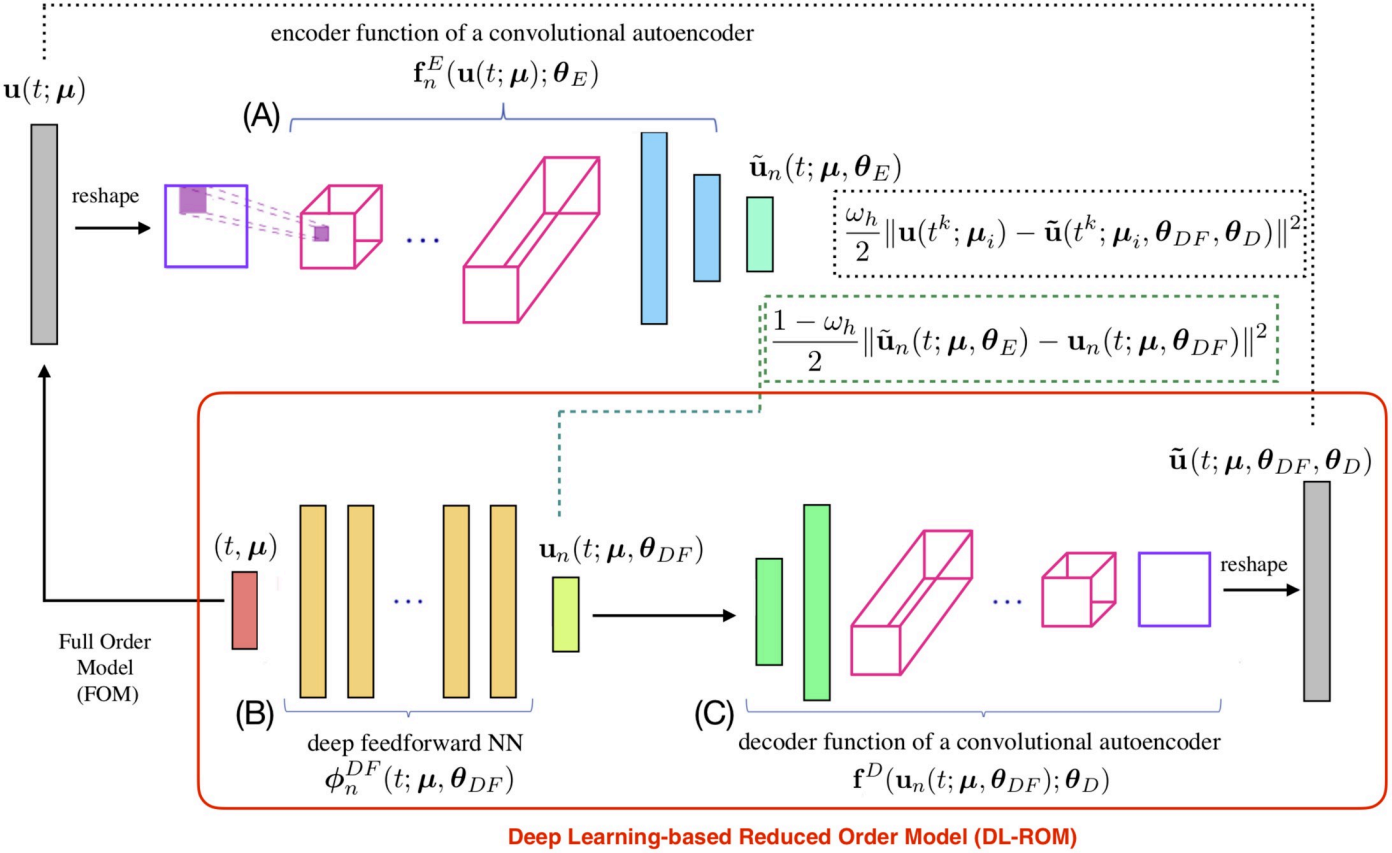
DL-ROM architecture. DL-ROM architecture used during the training phase. In the red box, the DL-ROM to be queried for any new selected couple (*t*, ***μ***) during the testing phase. The FOM solution **u**(*t*; ***μ***) is provided as input to block (A) which outputs ${\tilde{u}}_{n}\left(t;\mu \right)$. The same parameter instance associated to the FOM, i.e. (*t*; ***μ***), enters block (B) which provides as output **u**_*n*_(*t*; ***μ***) and the error between the low-dimensional vectors (dashed green box) is accumulated. The intrinsic coordinates **u**_*n*_(*t*; ***μ***) are given as input to block (C) returning the ROM approximation $\tilde{u}\left(t;\mu \right)$. Then the reconstruction error (dashed black box) is computed.

The architecture of DL-ROM is the one shown in Fig 1. The encoder function is used only during the training and validation steps; it is instead discarded during the testing phase. See [26] for further algorithmic details about the training and the testing algorithms required to build and evaluate a DL-ROM.

We highlight that the DL-ROM technique does not require to solve a (reduced) nonlinear dynamical system for the reduced degrees of freedom as in (7); rather, it evaluates a nonlinear map for any given couple (*t*, ***μ***_*test*_), for each *t* ∈ (0, *T*). Numerical results are extremely accurate, the mean relative error is indeed below 1% (see, e.g., Test 2), even if the biophysical dynamics underlying Eq (1) Moreover, the map features an extremely low dimension, in the most favorable scenario equal to *n*_***μ***_ + 1. From a computational perspective, remarkable gains and simplifications can be obtained against a linear ROM, since *(i)* no hyper-reduction is required to enhance the evaluation of any ***μ***- or **u**-dependent quantity, and *(ii)* even more interestingly, there is no need to evaluate the dynamics of the recovery variable *w* if one is only interested in the electrical potential.

## Results and discussion

We now assess the computational performances of the proposed DL-ROM strategy on four relevant test cases in cardiac electrophysiology. Our choice of the numerical tests is aimed at highlighting the performance of our DL-ROM method in challenging electrophysiology problems, namely pathological cases in portion of cardiac tissues or physiological scenarios on realistic left ventricle geometries.

The architecture used to perform all the numerical tests is the one reported in the S2 Appendix. To solve the optimization problem (13) and (14) we use the ADAM algorithm [47] with a starting learning rate equal to *η* = 10^−4^. Moreover, we perform cross-validation by splitting the data in training and validation and following a proportion 8:2 and we implement an early-stopping regularization technique to reduce overfitting [44].

To evaluate the performance of the DL-ROM, we use the loss function (14) and an error indicator defined as

$$\begin{array}{c} \epsilon_{rel}=\frac{1}{N_{test}}\sum_{i=1}^{N_{test}}(\frac{\sqrt{\sum_{k=1}^{N_{t}}\left|\right|u^{k}\left(\mu_{test,i}\right)-{\tilde{u}}^{k}\left(\mu_{test,i}\right){\left|\right|}^{2}}}{\sqrt{\sum_{k=1}^{N_{t}}\left|\right|u^{k}\left(\mu_{test,i}\right){\left|\right|}^{2}}}). \end{array}$$ (15)

Neural networks required by our DL-ROM technique have been implemented by means of the Tensorflow deep learning framework [48]. The training phase has been carried out on a workstation equipped with an Nvidia GeForce GTX 1070 8 GB GPU while, in addition to this hardware, the testing phase has also been carried out on a HPC cluster.

### Test 1: Two-dimensional slab with ischemic region

We consider the computation of the transmembrane potential in a square slab Ω = (0, 10 cm)^2^ of cardiac tissue in presence of an ischemic (non-conductive) region. The ischemic region may act as anatomical driver of cardiac arrhythmias like tachycardias and fibrillations. The system we want to solve is a slight modification of Eq (1), accounting for the presence of a non-conductive region which affects both the conductivity tensor and the ionic current term. The ischemic portion of the domain is modeled by replacing the conductivity tensor **D**(**x**), defined in (2), with $\bar{D}\left(x;\mu \right)=\sigma \left(x,\mu \right)D\left(x\right)$, where the function *σ*(**x**, ***μ***) is given by

$$\begin{array}{c} \begin{array}{cc} & \sigma \left(x;\mu \right)=\rho \left(x;\mu \right)+\sigma_{0}\left(1-\rho \left(x;\mu \right)\right),\\ & \rho \left(x;\mu \right)=1-\exp(-\frac{{\left(x_{1}-\mu_{1}\right)}^{4}+{\left(x_{2}-\mu_{2}\right)}^{4}}{2\alpha^{2}}). \end{array} \end{array}$$ (16)

In this case, *n*_*μ*_ = 2 parameters are considered, representing the coordinates of the center of the scar, belong to the parameter space $P={\left[3.5,6.5\;cm\right]}^{2}$. Moreover, *α* = 7 cm^2^, *σ*_0_ = 10^−4^, the transversal and longitudinal conductivities are *σ*_*t*_ = 12.9 ⋅ 0.1 cm^2^/ms and *σ*_*l*_ = 12.9 ⋅ 0.2 cm^2^/ms, respectively, and **f**_0_ = (1, 0)^*T*^, meaning that the tissue fibers are parallel to the *x*−axis. Similarly, the ionic current *I*_*ion*_(*u*, *w*) in (1) is replaced by ${\bar{I}}_{ion}\left(u,w;\mu \right)=\rho \left(x;\mu \right)I_{ion}\left(u,w\right)$. The applied current takes the form

$$\begin{array}{c} I_{app}\left(x,t\right)=C\exp(-\frac{{\left||x|\right|}^{2}}{\beta })1_{\left[0,\bar{t}\right]}\left(\tilde{t}\right), \end{array}$$

where *C* = 100 mA, *β* = 0.02 cm^2^ and $\bar{t}=2$ ms, consisting in a Gaussian-shaped applied stimulus with support in a circle with radius almost equal to 3 cm. The parameters appearing in (3) are set to *K* = 8, *a* = 0.01, *b* = 0.15, *ε*_0_ = 0.002, *c*_1_ = 0.2, and *c*_2_ = 0.3, see [49]. The equations have been discretized in space through linear finite elements by considering *N* = 64×64 = 4096 grid points. For the time discretization and the treatment of nonlinear terms, we use a one-step, semi-implicit, first order scheme (see [25] for further details) by considering a time step Δ*t* = 0.1/12.9 over (0, *T*) with *T* = 400 ms.

For the training phase, we uniformly sample *N*_*t*_ = 1000 time instances over (0, *T*) and consider *N*_*train*_ = 49 training-parameter instances, with ***μ***_*train*_ = (3.5+ *i*0.5, 3.5 + *j*0.5), *i*, *j* = 0, …, 6. The maximum number of epochs is set equal to *N*_*epochs*_ = 10000, the batch size is *N*_*b*_ = 40 and, regarding the early-stopping criterion, we stop the training if the loss function does not decrease in 500 epochs. For the testing phase, *N*_*test*_ = 36 testing-parameter instances ***μ***_*test*_ = (3.75 + *i*0.5, 3.75 + *j*0.5), *i*, *j* = 0, …, 5, have been considered.

**Fig 2:**
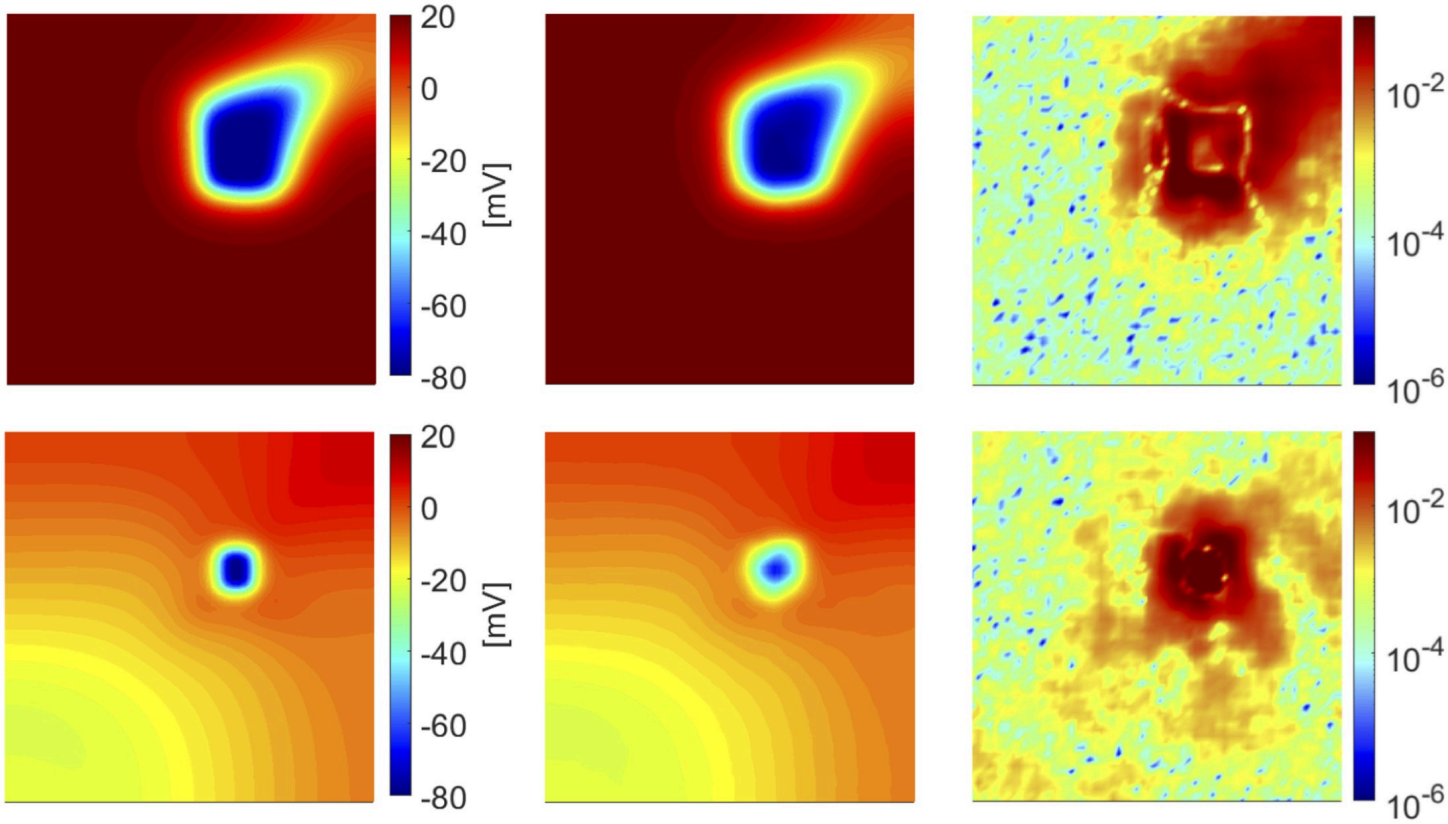
Test 1: Comparison between FOM and DL-ROM solutions for a testing-parameter instance. FOM solution (left), DL-ROM solution with *n* = 3 (center) and relative error ***ϵ***_*k*_ (right) for the testing-parameter instance ***μ***_*test*_ = (6.25, 6.25) cm at $\tilde{t}=100$ ms (top) and $\tilde{t}=356$ ms (bottom). The maximum of the relative error ***ϵ***_*k*_ is 10^−3^ and it is associated to the diseased tissue.

In Fig 2 we show the FOM and the DL-ROM solutions, the latter obtained with *n* = 3 for the testing-parameter instance ***μ***_*test*_ = (6.25, 6.25) cm at $\tilde{t}=100$ and 356 ms, respectively, together with the relative error $\epsilon_{k}\in R^{N}$, for *k* = 1, …, *N*_*t*_, defined as

$$\begin{array}{c} \epsilon_{k}=\frac{|u^{k}\left(\mu_{test}\right)-{\tilde{u}}^{k}\left(\mu_{test}\right)|}{\sqrt{\frac{1}{N_{t}}\sum_{k=1}^{N_{t}}\left|\right|u^{k}\left(\mu_{test}\right){\left|\right|}^{2}}}. \end{array}$$ (17)

While (15) is a synthetic indicator, the quantity defined in (17) is instead a function of the space independent variable. In Fig 2 (top) the tissue is depolarized except for the region occupied by scar and surrounding it, which is clearly characterized by a slower conduction. In Fig 2 (bottom) the tissue is starting to repolarize and even if the shape of the ischemic region is not sharply reproduced, the DL-ROM solution is able to capture the diseased (non-conductive) nature of this portion of tissue.

**Fig 3:**
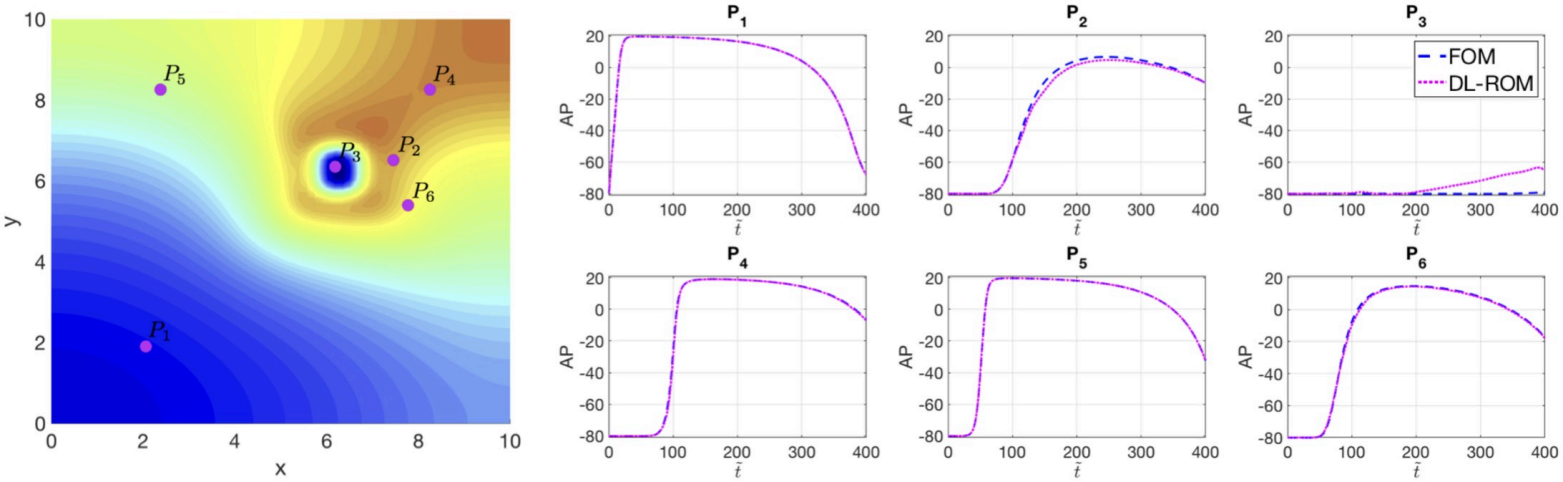
Test 1: Comparison between the FOM and DL-ROM APs at six points *P*_1_, …, *P*_6_. Left: FOM solution evaluated for ***μ**_test_* = (6.25, 6.25) cm at $\tilde{t}=400$ ms together with the points *P*_1_, …, *P*_6_. Right: APs evaluated for ***μ**_test_* = (6.25, 6.25) cm at points *P*_1_, …, *P*_6_. The DL-ROM, with *n* = 3, is able to to sharply reconstruct the AP in almost all the points and the main features are captured also for the points close to the scar.

In Fig 3 The DL-ROM is able to provide an accurate reconstruction of the AP at almost all points; the maximum error is associated to the point *P*_3_, the closest one to the center of the scar, for $\tilde{t}\geq 200$ ms. However, even in this case, the DL-ROM technique is able to capture the difference, in terms of AP peak values, between the diseased and the healthy tissue.

**Fig 4:**
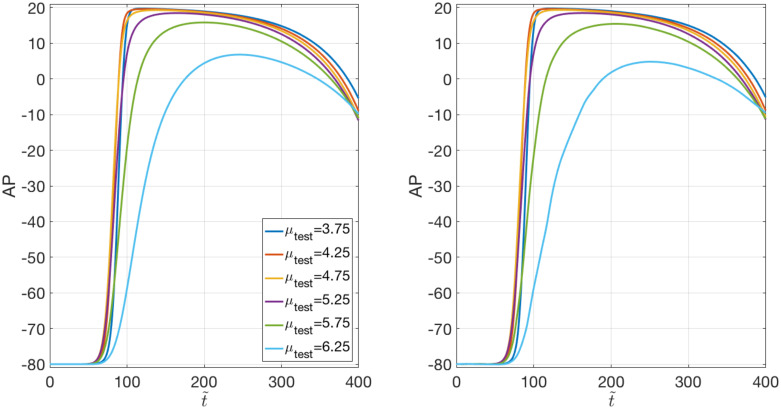
Test 1: Variability of the FOM and DL-ROM solutions over the parameter space. FOM (right) and DL-ROM (left) AP variability over P at *P* = (7.46, 6.51) cm. The DL-ROM sharply reconstructs the FOM variability over P.

The AP variability across the parameter space characterizing both the FOM and the DL-ROM solutions is shown in Fig 4. Still with a DL-ROM dimension *n* = 3, we report the APs for ***μ***_*test*_ = (*μ*_*test*_, *μ*_*test*_) cm, with *μ*_*test*_ = 3.75, 4.25, 4.75, 5.25, 5.75, 6.25, evaluated at *P* = (7.46, 6.51) cm. The DL-ROM is able to capture such variability over P; moreover, the larger ***μ***_*test*_, the smaller the distance between the point *P* and the scar, with their proximity impacting on the shape and the values of the AP. In particular, for *μ*_*test*_ = 6.25, the point *P* falls into the *grey zone*.

By using the DL-ROM technique and setting the dimension of the nonlinear trial manifold equal to the dimension of the solution manifold, i.e. *n* = 3, we obtain an error indicator (15) of *ϵ*_*rel*_ = 2.01 ⋅ 10^−2^. In order to assess the computational efficiency of DL-ROM, we compare it with the POD-Galerkin ROM relying on *N*_*c*_ local reduced bases; we report in Table 1 the maximum and minimum number of basis functions, among all the clusters, required by the POD-Galerkin ROM [24, 25] to achieve the same accuracy.

**Table 1:** Test 1: Dimensions of the POD-Galerkin ROM linear trial manifolds by varying the number of clusters.

*N*_*c*_ = 1	*N*_*c*_ = 2	*N*_*c*_ = 4	*N*_*c*_ = 6
250	219	200	193
	107	35	26

Maximum and minimum dimensions of the local reduced bases (that is, linear trial manifolds) built by the POD-Galerkin ROM for different numbers *N*_*c*_ of clusters.

**Fig 5:**
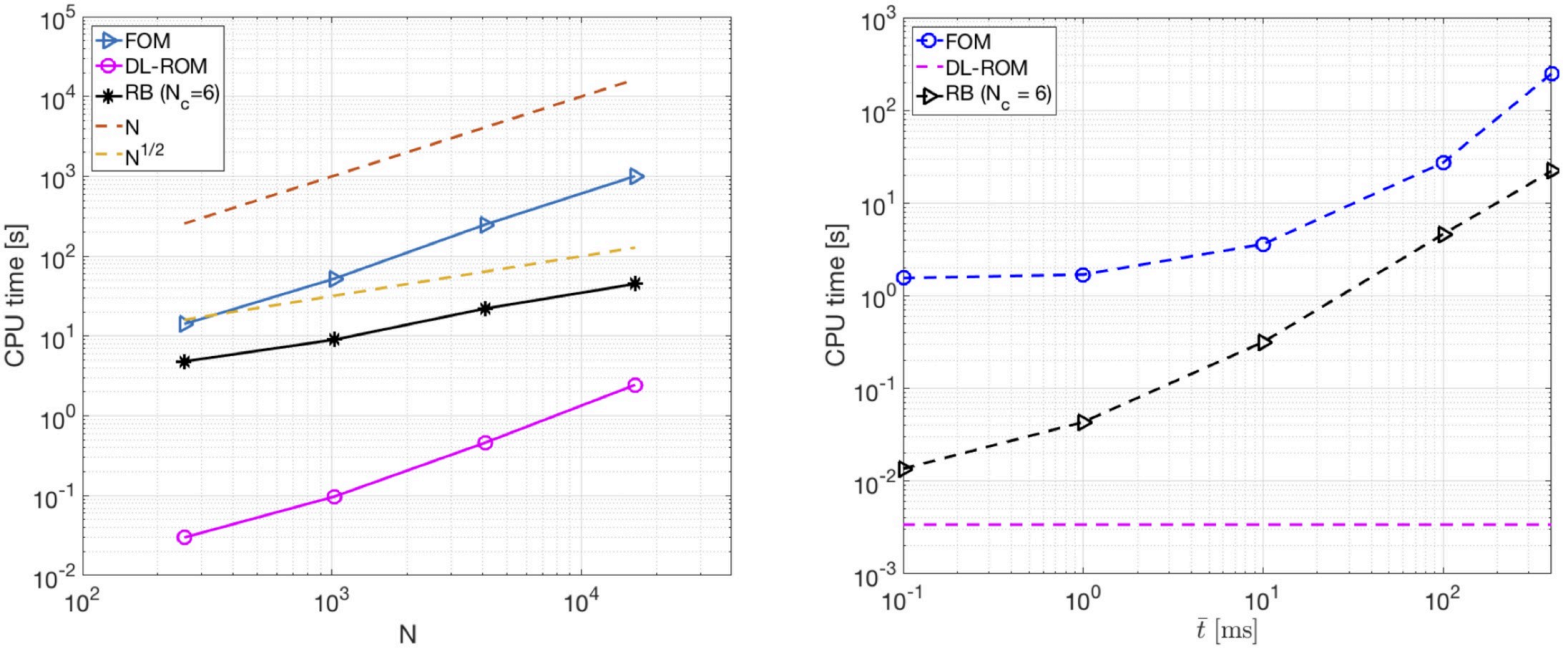
Test 1: FOM, DL-ROM and POD-Galerkin ROM CPU computational times. Left: CPU time required to solve the FOM, by DL-ROM at testing time with *n* = 3 and by the POD-Galerkin ROM at testing time with *N*_*c*_ = 6 vs. *N*. The DL-ROM provides the smallest testing computational time for each *N* considered. Right: FOM, POD-Galerkin ROM and DL-ROM CPU computational times to compute $\tilde{u}\left(\bar{t};\mu_{test}\right)$ vs. $\bar{t}$ averaged over the testing set. Thanks to the fact that the DL-ROM can be queried at any time istance it is extremely efficient in computing $\tilde{u}\left(\bar{t};\mu_{test}\right)$ with respect to both the FOM and the POD-Galerkin ROM.

In Fig 5 (left) we compare the CPU time required to solve the FOM (through linear finite elements) over the time interval (0, *T*), with the one needed by DL-ROM with *n* = 3, and the POD-Galerkin ROM with *N*_*c*_ = 6 local reduced bases, at testing time, by varying the FOM dimension *N*. Here, with testing time we refer, both for the DL-ROM and the POD-Galerkin ROM, to the time needed to query the ROM over the whole interval (0, *T*), by using for each technique the proper time resolution, for a given testing-parameter instance. Since the DL-ROM solution can be queried at a given time without requiring any solution of a dynamical system to recover the former time instances, the DL-ROM can employ larger time windows compared to the time steps required by the solution of the FOM and POD-Galerkin ROM dynamical systems for the cases at hand. This fact also has a positive impact on the data used during the training phase Indeed, in order to build the snapshot matrix, we uniformly sample *N*_*t*_ time instances of the FOM solution over *T*/Δ*t* = 4000 time steps; for each training parameter instance, only 25% of 4000 snapshots are retained from the FOM solution in the DL-ROM case, against 4000 snapshots in the POD-Galerkin ROM case. The speed-up obtained, for each value of *N* considered, is reported in Table 2 Both the DL-ROM and the POD-Galerkin ROM allow us to decrease the computational costs associated to the computation of the FOM solution for a testing-parameter instance. However, for a desired level of accuracy, CPU times required by the POD-Galerkin ROM during the testing phase are remarkably higher than the ones required by a DL-ROM with *n* = 3.

**Table 2:** Test 1: DL-ROM and POD-Galerkin ROM vs. FOM speed-up.

	*N* = 256	*N* = 1024	*N* = 4096	*N* = 16384
FOM vs. DL-ROM	472	536	539	412
FOM vs. POD-Galerkin ROM	3	6	12	22

DL-ROM and POD-Galerkin ROM vs. FOM speed-up by varying *N*. The DL-ROM speed-up is remarkably higher than the one obtained by using the POD-Galerkin ROM.

Both the DL-ROM and the POD-Galerkin ROM depend on the FOM dimension *N*. In the case of DL-ROM, the dependency on *N* at testing time, for a fixed value of Δ*t*, is due to the presence of the decoder function; indeed, the process of learning the reduced dynamics (and so the dimension of the nonlinear trial manifold) does not depend on the FOM dimension. On the other hand, the dependence of the POD-Galerkin ROM on the FOM dimension also impacts on the dimension of the local linear trial manifolds: in general, by increasing *N* the dimension of each local linear subspace also increases. Referring to Fig 5 (left) and Table 2, the CPU time required by the DL-ROM at testing time scales linearly with *N*, instead the one required by the POD-Galerkin ROM scales linearly with $\sqrt{N}$. In particular, even for the larger FOM dimension considered (*N* = 16384 for this test case), our DL-ROM is 19 times faster than the POD-Galerkin ROM. We are not able to run simulations for *N* > 16384, because of the limitation of the computing resources we have at our disposal. Despite the trend in Fig 5 (left) is apparently not favorable for the DL-ROM technique, practice indicates that the CPU time for DL-ROM is smaller than the one for the POD-Galerkin ROM for *small* values of *N*, in other words only with very large values of *N* the POD-Galerkin ROM outperforms the DL-ROM strategy. Indeed, a linear fitting of the DL-ROM and the POD-Galerkin ROM CPU times *N* = 65536 and *N* = 262144 for this test case represent FOM dimensions corresponding to mesh sizes *h* needed to solve, by means of linear finite elements, the problem on a 3D slab geometry both for physiological and pathological electrophysiology in the case a ten Tusscher-Panfilov ionic model [50] is used. This latter would indeed require smaller values of *h* compared to the Aliev-Panfilov model, due to the shape of the AP. See, e.g., [51, 52] for further details. in Fig 5 (left) highlights that for *N* = 65536 and *N* = 262144, DL-ROM could be almost 10 and 5 times, respectively, faster than the POD-Galerkin ROM for the same values of *N*. Note that the results of this section have been obtained by employing the DL-ROM on a single CPU, an architecture which is not favorable to neural networks Indeed, all tests are performed on a node (20 Intel^®^ Xeon^®^ E5-2640 v4 2.4GHz cores), using 5 cores, of our in-house HPC cluster. Further improvements are expected when employing our DL-ROM on a GPU for a given testing-parameter instance.

We highlight that since the DL-ROM solution can be evaluated at any desired time instance without *solving* any dynamical system, the resulting computational time entailed by the DL-ROM at testing time are drastically reduced compared to the ones required by the FOM or the POD-Galerkin ROM to compute solutions at a particular time instance. In Fig 5 (right) we show the DL-ROM, FOM and POD-Galerkin ROM CPU time needed to compute the approximated solution at $\bar{t}$, for $\bar{t}=1$, 10, 100 and 400 ms averaged over the testing set and with *N* = 4096. We perform the training phase of the POD-Galerkin ROM over the original time interval (0, *T*) ms and we report the results for *N*_*c*_ = 6, the number of clusters for which the smallest computational time is obtained. The DL-ROM CPU time to compute $\tilde{u}\left(\bar{t};\mu_{test}\right)$ does not vary over $\bar{t}$ and, by choosing $\bar{t}=T$, the DL-ROM speed-ups are equal to 7.3 × 10^4^ and 6.5 × 10^3^ with respect to the FOM and the POD-Galerkin ROM, with *N*_*c*_ = 6, computational times.

Regarding the training (offline) times, in the case of a FOM dimension *N* = 4096, training the DL-ROM neural network on a GTX 1070 8GB GPU requires about 21 hours, whereas training the POD-Galerkin ROM (with *N*_*c*_ = 6 local bases) on 5 cores of a node of the HPC cluster at our disposal requires about 3 hours; in both the cases, the time needed to assemble the snapshot matrix **S** is not included. However, the 7 times higher training time of the DL-ROM is justified by the efficiency gained at testing time; indeed, a query to the DL-ROM online requires 0.08 seconds on a GPU, implying a speedup of about 275 times compared to the POD-Galerkin ROM.

### Test 2: Two-dimensional slab with figure of eight re-entry

The most recognized cellular mechanisms sustaining atrial tachycardia is re-entry [53]. The particular kind of re-entry we deal with in this test case is called *figure of eight* re-entry, and can be obtained by solving Eq (1). To induce the re-entry, we apply a classical S1-S2 protocol [3, 54]. In particular, we consider a square slab of cardiac tissue Ω = (0, 2 cm)^2^ and apply an initial stimulus (S1) at the bottom edge of the domain, i.e.

$$\begin{array}{c} I_{app}^{1}\left(x,t\right)=1_{\Omega_{1}}\left(x\right)1_{\left[t_{1}^{i},t_{1}^{f}\right]}\left(\tilde{t}\right), \end{array}$$ (18)

where Ω_1_ = {**x** ∈ Ω: *y* ≤ 0.1}, $t_{1}^{i}=0$ ms and $t_{1}^{f}=5$ ms. A second stimulus (S2) under the form

$$\begin{array}{c} I_{app}^{2}\left(x,t;\mu \right)=1_{\Omega_{2}\left(\mu \right)}\left(x\right)1_{\left[t_{2}^{i},t_{2}^{f}\right]}\left(\tilde{t}\right), \end{array}$$ (19)

with Ω_2_(*μ*) = {**x** ∈ Ω: (*x* − 1)^2^ + (*y* − *μ*)^2^ ≤ (0.2)^2^}, $t_{2}^{i}=70$ ms and $t_{2}^{f}=75$ ms, is then applied. Here the parameter *μ* is the *y*-coordinate of the center of the second circular stimulus. We analyze two configurations: *(i)* a first case in which both re-entry and non re-entry cases are generated, by considering P=[0.5,1.1] cm; *(ii)* a second case in which instead only re-entrant dynamics are taken into account, and P=[0.8,1.1] cm. These choices have been made to obtain a re-entry elicited and sustained until *T* = 175 ms. Moreover, we restrict ourselves to the time interval [95, 175] ms, without considering the time window [0, 95) ms in which the re-entry has not arisen yet, and is common to all *μ* instances. The time step is Δ*t* = 0.2/12.9. We consider *N* = 256 × 256 = 65536 grid points, implying a mesh size *h* = 0.0784 mm; this mesh size is recognized to correctly solve the tiny transition front developing during depolarization of the tissue, see [51, 52]. The fibers are parallel to the *x*-axis and the conductivities in the longitudinal and transversal directions to the fibers are *σ*_*l*_ = 2 × 10^−3^ cm^2^/ms and *σ*_*t*_ = 3.1 × 10^−4^ cm^2^/ms, respectively. The parameters appearing in (3) are set to *K* = 8, *a* = 0.1, *b* = 0.1, *ε*_0_ = 0.01, *c*_1_ = 0.14, and *c*_2_ = 0.3, see [55].

The snapshot matrix is built by solving problem (1), completed with the applied currents (18) and (19), by means of a semi-implicit scheme, over *N*_*t*_ = 400 time instances. Moreover, we consider *N*_*train*_ = 13 training-parameter instances uniformly distributed in the parameter space and *N*_*test*_ = 12 testing-parameter instances, each of them corresponding to the midpoint of two consecutive training-parameter instances. The maximum number of epochs is set equal to *N*_*epochs*_ = 6000, the batch size is *N*_*b*_ = 3, due to the high GPU memory occupation of each sample. Regarding the early-stopping criterion, we stop the training if the loss does not decrease in 1000 epochs.

**Fig 6:**
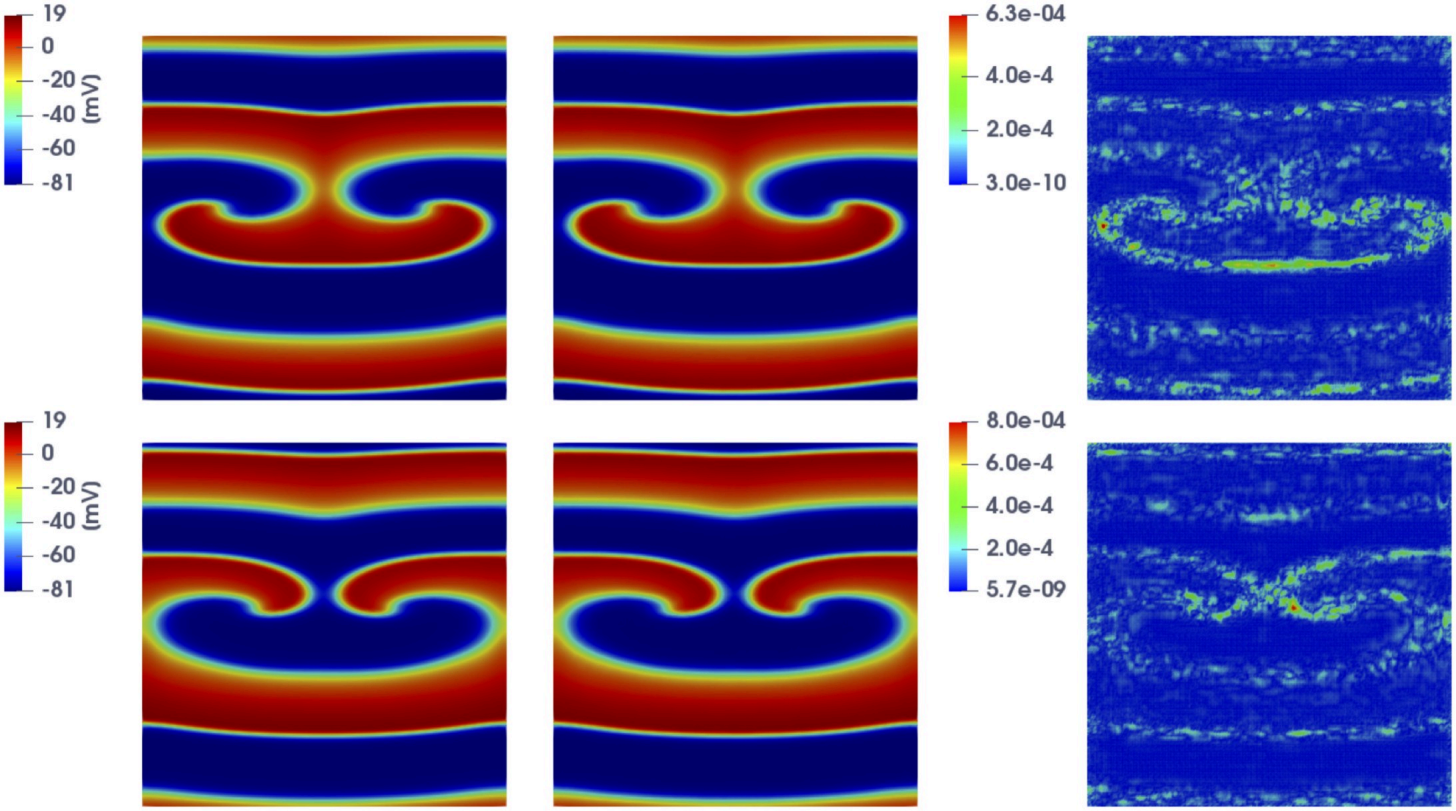
Test 2: Comparison between FOM and DL-ROM solutions for a testing-parameter instance. FOM solution (left), DL-ROM one (center) with *n* = 5, and relative error ***ϵ***_*k*_ (right) at $\tilde{t}=141.2$ ms (top) and $\tilde{t}=157.2$ ms (bottom), for the testing-parameter instance *μ*_*test*_ = 0.9625 cm. The relative error ***ϵ***_*k*_ is below 0.1% at both time instants.

In Fig 6 we show the FOM solution and the DL-ROM one obtained by setting the reduced dimension to *n* = 5, for the testing-parameter instance *μ*_*test*_ = 0.9625 cm, at $\tilde{t}=141.2$ ms and $\tilde{t}=157.2$ ms, together with the relative error $\epsilon_{k}\in R^{N}$ computed according to (17).

We introduce the relative error $\epsilon_{k}^{s}\in R^{N}$, for *k* = 1, …, *N*_*t*_, given by

$$\begin{array}{c} \epsilon_{k}^{s}=\frac{|u^{k}\left(\mu_{test}\right)-{\tilde{u}}^{k}\left(\mu_{test}\right)|}{\|u^{k}\left(\mu_{test}\right){\|}_{1}}\times 100. \end{array}$$ (20)

**Fig 7:**
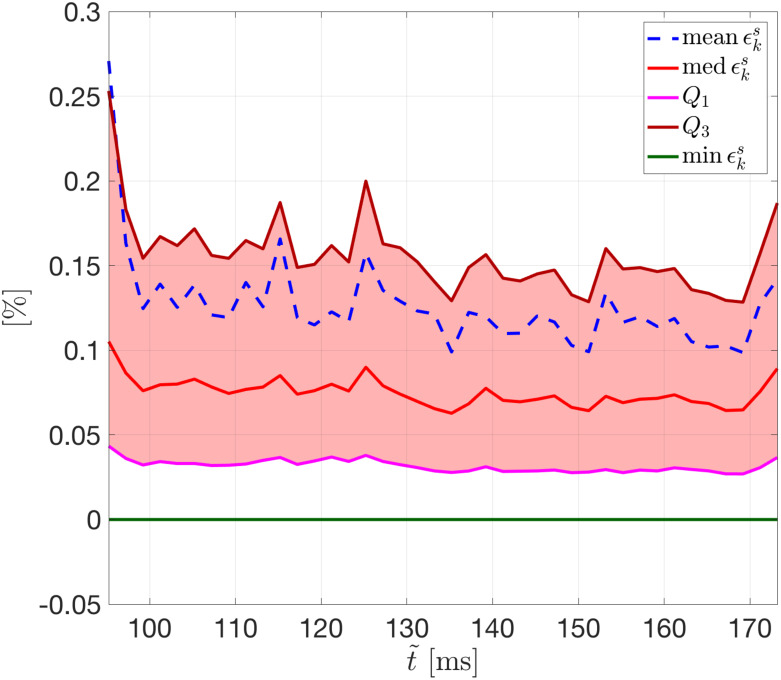
Test 2: Trend of the relative error over time. Relative error $\epsilon_{k}^{s}$ vs. $\tilde{t}$ with *n* = 5 for the testing-parameter instance *μ*_*test*_ = 0.9625 cm (the red band indicates the IQR). The error distribution is almost uniform over time.

The trend of (20) over time, for the selected testing-parameter instance *μ*_*test*_ = 0.9625 cm, is depicted in Fig 7; we highlight that the error is, on average, always smaller than 0.3%. In particular, in Fig 7 we show the mean, the median, and the first and third quartile (all computed with respect to the spatial coordinates) of the relative error, as well as its minimum. The interquartile range (IQR) shows that the distribution of the error is almost uniform over time, and that the maximum error is associated to the first time instant—this latter being the time instant at which the solution is most different over P.

**Fig 8:**
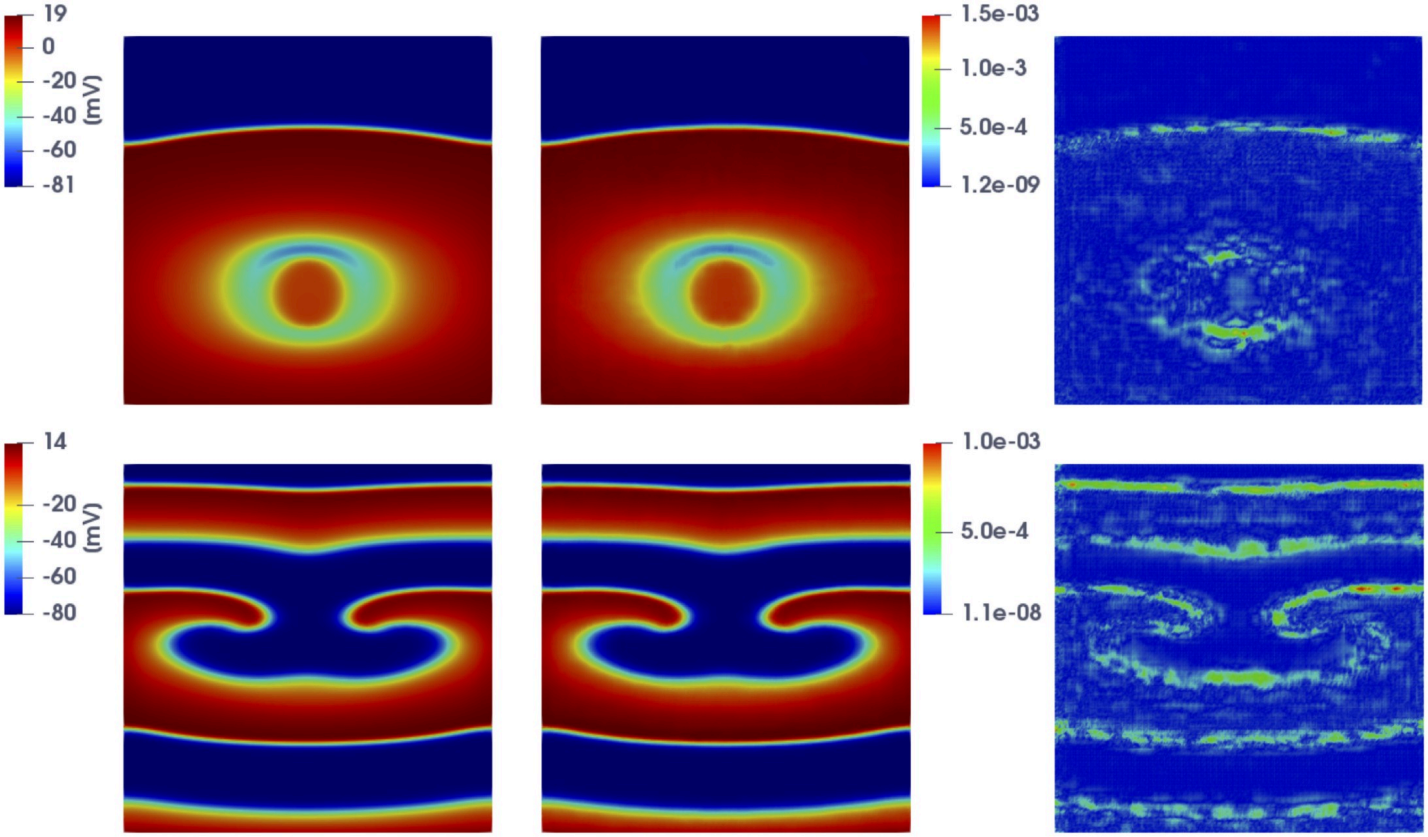
Test 2: Comparison between FOM and DL-ROM solutions for different testing-parameter instances. FOM solution (left), DL-ROM one (center) with *n* = 5, and relative error ***ϵ***_*k*_ (right) at $\tilde{t}=153.2$ ms, for the testing-parameter instance *μ*_*test*_ = 0.6125 cm (top) and *μ*_*test*_ = 0.9125 cm (bottom). In both cases the relative error ***ϵ***_*k*_ is below 1%.

In Fig 8 we show the FOM and the DL-ROM solutions, the latter obtained by setting *n* = 5, for the last time instance, i.e. at $\tilde{t}=153.2$ ms, for *μ*_*test*_ = 0.6125 cm and *μ*_*test*_ = 0.9125 cm, in order to point out the variability of the solution over the parameter space P=[0.5,1.1] cm and the ability of DL-ROM to capture it. In particular, in Fig 8 we compare the FOM and the DL-ROM solutions for two testing-parameter instances corresponding to *(i)* a case in which the re-entry does not arise (top), since S2 is too far from the front elicited with S1, i.e. the tissue around S2 is no longer in the refractory period and is able to activate again; *(ii)* a case in which the re-entry is elicited, the electrical signal follows an alternative circuit looping back upon itself and developing a self-perpetuating rapid and abnormal activation (bottom).

**Fig 9:**
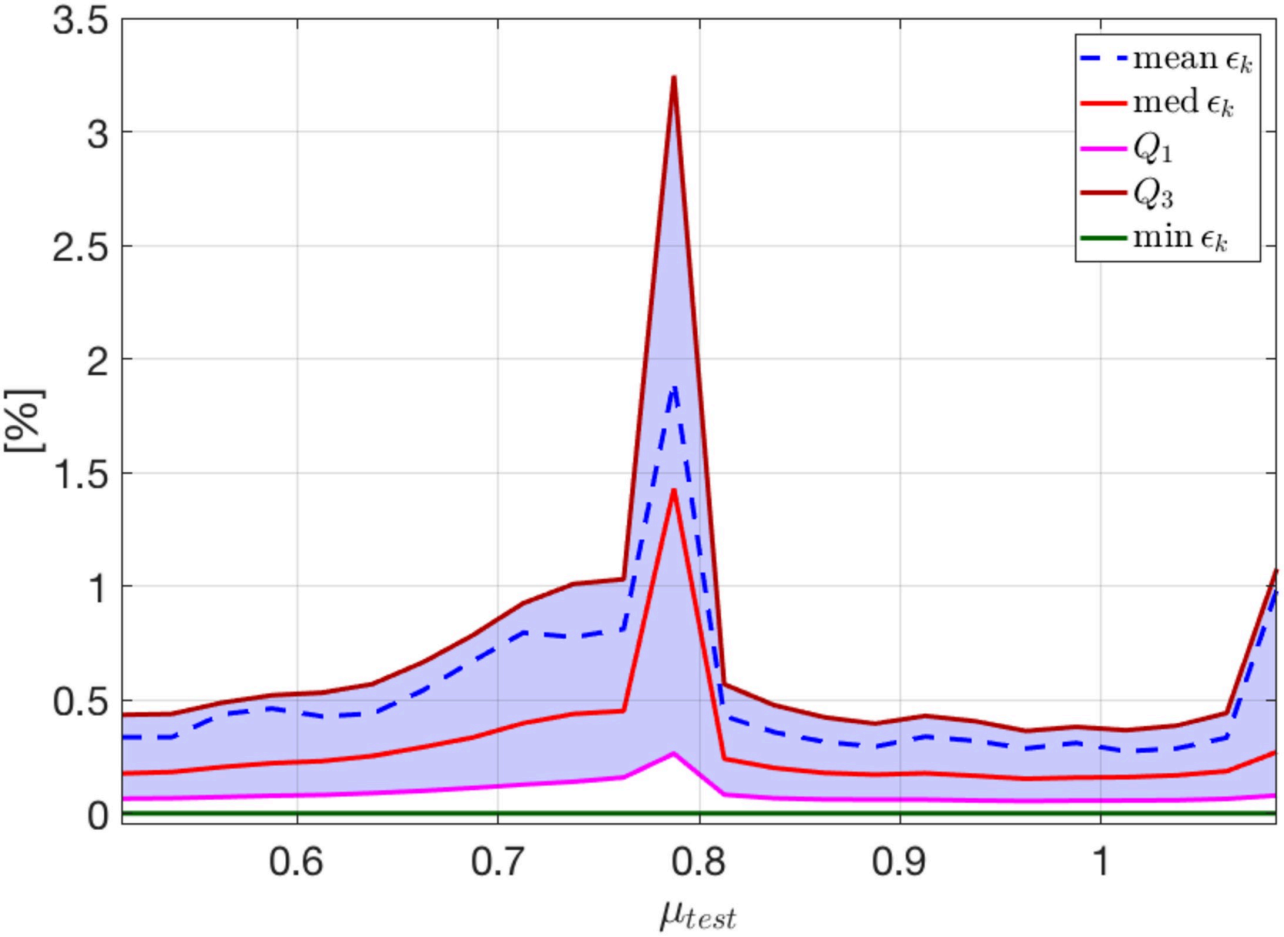
Test 2: Trend of the relative error over the parameter space. Relative error $\epsilon_{k}^{s}$ vs. *μ*_*test*_ with *n* = 5 for the time instance $\tilde{t}=147$ ms (the violet band indicates the IQR). The maximum error is associated to *μ*_*test*_ = 0.7875 cm, the testing-parameter instance between *μ*_*train*_ = 0.775 cm (the last value for which re-entry does not arise) and *μ*_*train*_ = 0.8 cm (the first value for which re-entry is elicited).

In Fig 9 we show the trend of the relative error (20) at a selected time instance given by *t* = 147 ms over the parameter space, reporting the mean, the median, the first and third quartile, as well as its minimum (all computed with respect to the spatial coordinates). We highlight that the error is always smaller than 1%, except for its maximum which is associated to the value of *μ_test_* corresponding to the transition between re-entry and non re-entry dynamics.

**Fig 10:**
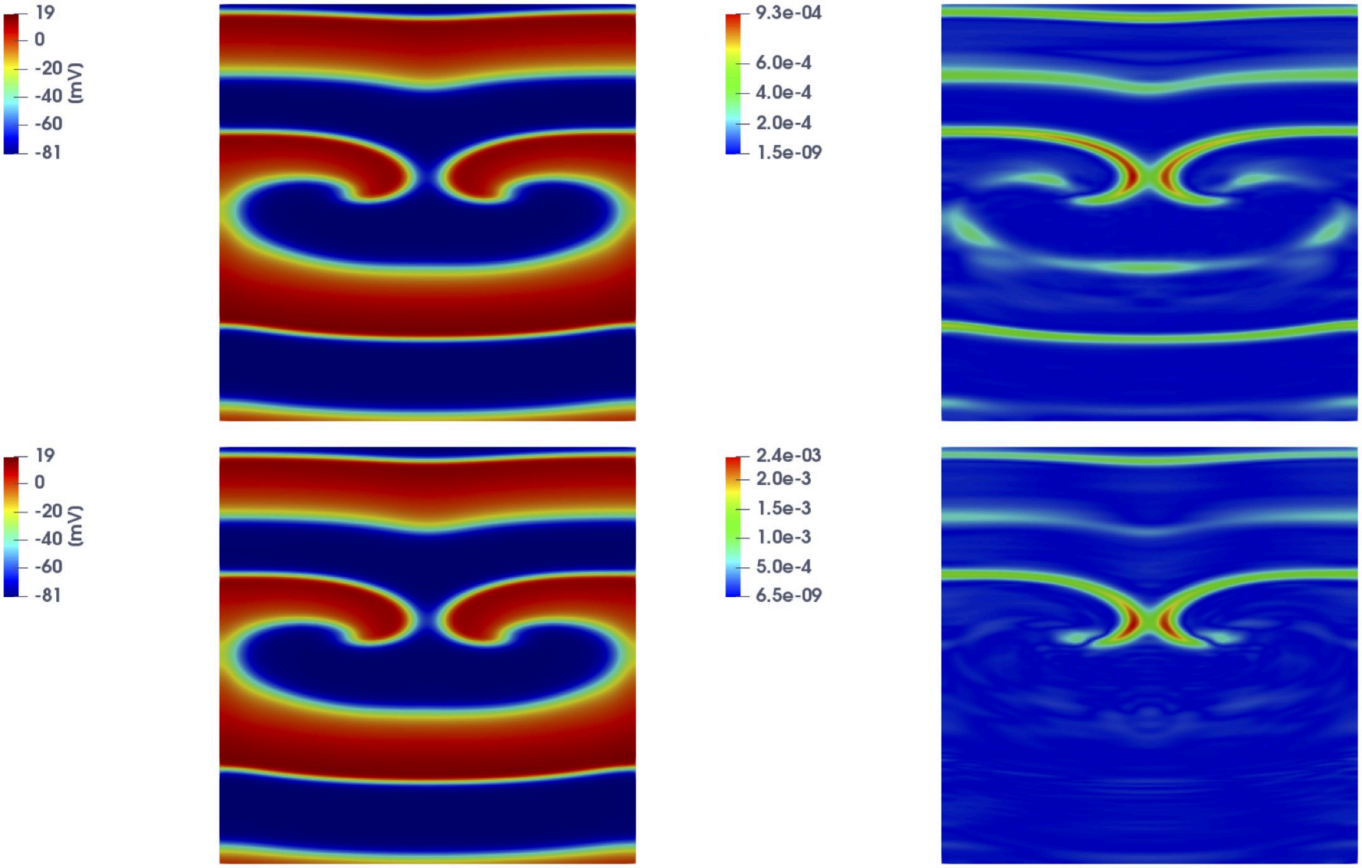
Test 2: POD-Galerkin ROM solutions for different testing-parameter instances. POD-Galerkin ROM solution (left) and relative error ***ϵ***_*k*_ (right) for *N*_*c*_ = 2 (top) and *N*_*c*_ = 4 (bottom) at $\tilde{t}=157.2$ ms, for *μ*_*test*_ = 0.9625 cm.

Let us now focus on the case in which only re-entrant dynamics are generated, and P=[0.8,1.1] cm, in order to compare the FOM, the POD-Galerkin ROM and the DL-ROM approximations. In Fig 10 we show the solutions obtained through the POD-Galerkin ROM with *N*_*c*_ = 2 (top) and *N*_*c*_ = 4 (bottom) local reduced bases, along with the relative error defined in (17), for the testing-parameter instance *μ*_*test*_ = 0.9625 cm at $\tilde{t}=157.2$ ms. In both cases, we have considered the setting yielding the most efficient POD-Galerkin ROM approximation, which require about 30 (40, respectively) seconds to be evaluated. By comparing Figs 10 and 6 (bottom), we observe that the DL-ROM outperforms the POD-Galerkin ROM in terms of accuracy.

**Fig 11:**
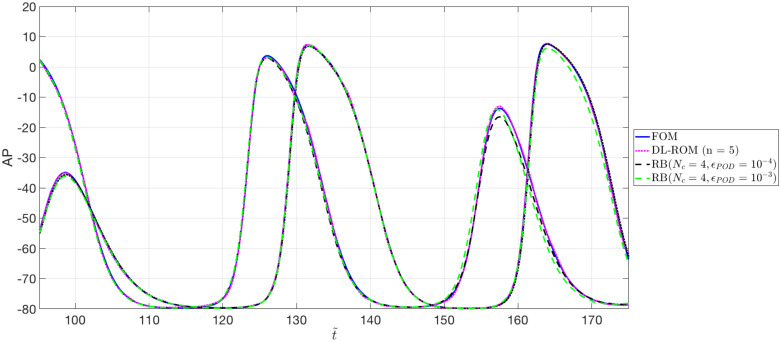
Test 2: FOM, DL-ROM and POD-Galerkin ROM APs at P_1_ and P_2_. AP obtained through the FOM, the DL-ROM and the POD-Galerkin ROM with *N*_*c*_ = 4, for the testing-parameter instance *μ*_*test*_ = 0.9625 cm, at *P*_1_ = (0.64, 1.11) cm and *P*_2_ = (0.69, 1.03) cm. The POD-Galerkin ROM approximations are obtained by imposing a POD tolerance *ε*_*POD*_ = 10^−4^ and 10^−3^, resulting in error indicator (15) values equal to 5.5 × 10^−3^ and 7.6 × 10^−3^, respectively.

In Fig 11 we show the action potentials obtained through the FOM, the DL-ROM and the POD-Galerkin ROM (with *N*_*c*_ = 4 local reduced bases), for the testing-parameter instance *μ*_*test*_ = 0.9625 cm, evaluated at *P*_1_ = (0.64, 1.11) cm and *P*_2_ = (0.69, 1.03) cm. These two points are close to the left core of the figure of eight re-entry, where a shorter action potential duration, and lower peak values of AP, with respect to a healthy case, due to the meandering of the cores, are observed. The AP dynamics at those points is accurately captured by the DL-ROM, while the POD-Galerkin ROM leads to slightly less accurate results requiring larger testing times.

We now compare the computational times required by the FOM, the POD-Galerkin ROM (for different values of *N*_*c*_) and the DL-ROM, keeping for all the same degree of accuracy achieved by DL-ROM, i.e. *ϵ*_*rel*_ = 7.87 × 10^−3^, and running the code on the hardware each implementation is optimized for—a CPU for the FOM and the POD-Galerkin ROM, a GPU Indeed, at each layer of a neural network thousands of identical computations must be performed. The most suitable hardware architectures to carry out this kind of operations are GPUs because (i) they have more computational units (cores) and (ii) they have a higher bandwidth to retrieve from memory. Moreover, in applications requiring image processing, as CNNs, the graphics specific capabilities can be further exploited to speed up calculations. for the DL-ROM. In Table 3 we report the CPU time needed to compute the FOM solution and the POD-Galerkin ROM approximation (online, at testing phase), both on a full 64 GB node (20 Intel^®^ Xeon^®^ E5-2640 v4 2.4GHz cores), and the GPU time required by the DL-ROM to compute 875 time instances (the same number of time instants considered in the solution of the dynamical systems associated to the FOM and the POD-Galerkin ROM) at testing time, by fixing its dimension to *n* = 5, on an Nvidia GeForce GTX 1070 8 GB GPU. For the sake of completeness, we also report the computational time required by the DL-ROM when employing a single CPU node. It is evident that a POD-Galerkin ROM, built employing a global reduced basis (*N*_*c*_ = 1), is not amenable to a complex and challenging pathological cardiac electrophysiology problem like the figure of eight re-entry. Using a nonlinear approach, for which the solution manifold is approximated through a piecewise linear trial manifold (as in the case of *N*_*c*_ = 2 or *N*_*c*_ = 4 local reduced bases) reduces the online computational time. However, the DL-ROM still confirms to provide a more efficient ROM, almost 5 (or 2) times faster on the CPU, and 39 (or 19) faster on the GPU, than the POD-Galerkin ROM with *N*_*c*_ = 2 (or *N*_*c*_ = 4) local reduced bases.

**Table 3:** Test 2: FOM, POD-Galerkin ROM and DL-ROM computational times.

	time [s]	FOM/ROM dimensions
FOM (CPU)	382	*N* = 65536
DL-ROM (CPU/GPU)	15/1.2	*n* = 5
POD-Galerkin ROM *N*_*c*_ = 1 (CPU)	103	*n* = 1538
POD-Galerkin ROM *N*_*c*_ = 2 (CPU)	70	*n* = 1158, 751
POD-Galerkin ROM *N*_*c*_ = 4 (CPU)	33	*n* = 435, 365, 298, 45

POD-Galerkin ROM and DL-ROM computational times along with FOM and reduced trial manifold(s) dimensions. DL-ROM provides a more efficient ROM with respect to the POD-Galerkin ROMs.

**Fig 12:**
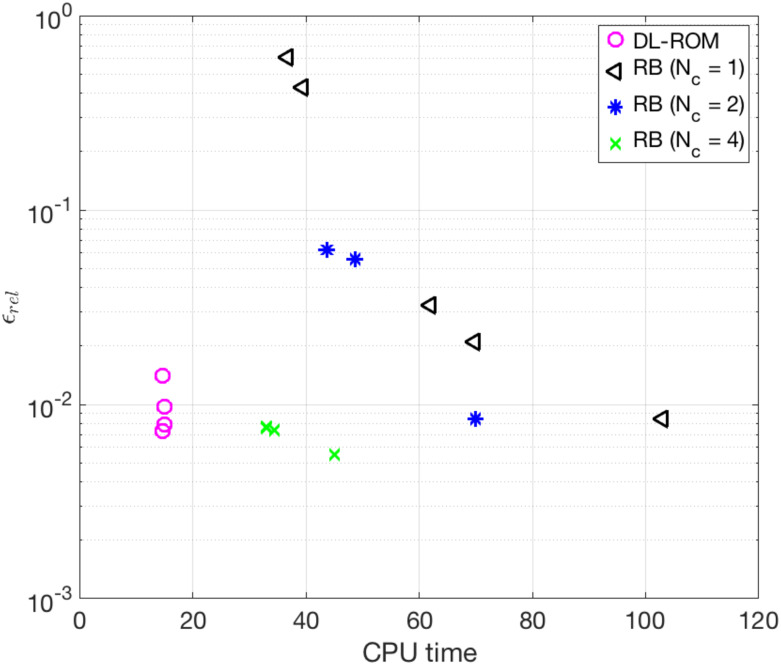
Test 2: Trend of the error indicator versus the CPU testing computational time. Error indicator *ϵ*_*rel*_ vs. CPU testing computational time for different values of *N*_*c*_ and *ε*_*POD*_. The DL-ROM outperforms the POD-Galerkin ROM in terms of both efficiency and accuracy.

In Fig 12 we show the trend of the error indicator (15) over the testing set versus the CPU time both for the DL-ROM and the POD-Galerkin ROM at testing phase. Slight improvements of the performance of DL-ROM, in terms of accuracy, are obtained for a small increase of the DL-ROM dimension *n*, coherently with our previous findings reported in [26]. Indeed, the DL-ROM is able, also in this case, to accurately represent the solution manifold by a reduced nonlinear trial manifold of dimension *n*_*μ*_ + 1 = 2; for the case at hand, we report the results for *n* = 5 (very close to the intrinsic dimension *n*_*μ*_ + 1 = 2 of the problem, and much smaller than the POD-Galerkin ROM dimension), providing slightly smaller values of the error indicator (15) than in the case *n* = 2. Regarding instead the POD-Galerkin ROM, in Fig 12 we report results obtained for different tolerances *ε*_*POD*_ = 10^−4^, 5 ⋅ 10^−4^, 10^−3^, 5 ⋅ 10^−3^, 10^−2^. In the cases *N*_*c*_ = 2 and *N*_*c*_ = 4 we only report the results related to the smallest POD tolerances, which indeed allow us to meet the prescribed accuracy on the approximation of the gating variable, which would otherwise impact dramatically on the overall accuracy of the POD-Galerkin ROM. Moreover, we do not consider more than *N*_*c*_ = 4 local reduced bases in order not to generate too small local linear subspaces, which would be otherwise unable to approximate the variability of the solution over the parameter space P accurately. Indeed, by considering a larger number of clusters, the dimension of some linear subspaces becomes so small that the error would start to increase compared to the one obtained with fewer clusters. As shown in Fig 12, the proposed DL-ROM outperforms the POD-Galerkin ROM in terms of both efficiency and accuracy.

Regarding the training (offline) times, in the case of a FOM dimension *N* = 4096, training the DL-ROM neural network on a GTX 1070 8GB GPU requires about 64 hours, whereas training the POD-Galerkin ROM (with *N*_*c*_ = 4 local bases) on a full node (20 Intel^®^Xeon^®^ E5-2640 v4 2.4GHz cores) of a HPC cluster requires about 4 hours; in both cases, the time needed to assemble the snapshot matrix **S** is not included. The DL-ROM training time is related to the value chosen during the hyper-parameters tuning for the batch size, i.e. *N*_*b*_ = 3; indeed we highlight that by choosing a slightly higher value of *N*_*b*_, it is possible to decrease the GPU computational training time as long as we look for a lower accuracy. The fact that the DL-ROM training time is 16 times higher than the POD-Galerkin ROM one is again justified by the efficiency introduced at testing time. Indeed, a query to the DL-ROM online requires 1.2 seconds on a GPU, implying a speedup of about 28 times compared to the POD-Galerkin ROM.

### Test 3: Three-dimensional ventricle geometry

We consider the solution of the coupled system (1) in a three-dimensional left ventricle (LV) geometry, obtained from the 3D Human Heart Model provided by Zygote [56]. Here, we consider a single (*n*_*μ*_ = 1) parameter, given by the longitudinal conductivity in the fibers direction. The conductivity tensor takes the form

$$\begin{array}{c} D\left(x;\mu \right)=\sigma_{t}I+\left(\mu -\sigma_{t}\right)f_{0}⊗f_{0}, \end{array}$$ (21)

where *σ*_*t*_ = 12.9 ⋅ 0.02 mm^2^/ms; **f**_0_ is determined at each mesh point through a *rule-based* approach, by solving a suitable Laplace problem [57]. The resulting fibers field is reported in S2 File. The applied current is defined as

$$\begin{array}{c} I_{app}\left(x,t\right)=\frac{C}{{\left(2\pi \right)}^{3/2}\alpha }\exp(-\frac{||x-\bar{x}{\left|\right|}^{2}}{2\beta })1_{\left[0,\bar{t}\right]}\left(\tilde{t}\right), \end{array}$$

where $\bar{t}=2$ ms, *C* = 1000 mA, *α* = 50, *β* = 50 mm^2^, $\bar{x}={\left[44.02,1349.61,63.28\right]}^{T}$ mm.

In order to build the snapshot matrix ***S***, we solve problem (1) completed with the conductivity tensor (21) by means of linear finite elements, on a mesh made by *N* = 16365 vertices, and a semi-implicit scheme in time over a uniform partition of (0, *T*) with *T* = 300 ms and time step Δ*t* = 0.1/12.9. We uniformly sample *N*_*t*_ = 1000 time instances in (0, *T*) and we zero-padded [44] the snapshot matrix to reshape each column in a 2D square matrix. The parameter space is provided by P=12.9·[0.04,0.4] mm^2^/ms; here we consider *N*_*train*_ = 25 training-parameter instances and *N*_*test*_ = 24 testing-parameter instances computed as in Test 2. In this case, the maximum number of epochs is set to *N*_*epochs*_ = 30000, the batch size is *N*_*b*_ = 40 and the training is stopped if the loss does not decrease over 4000 epochs.

**Fig 13:**
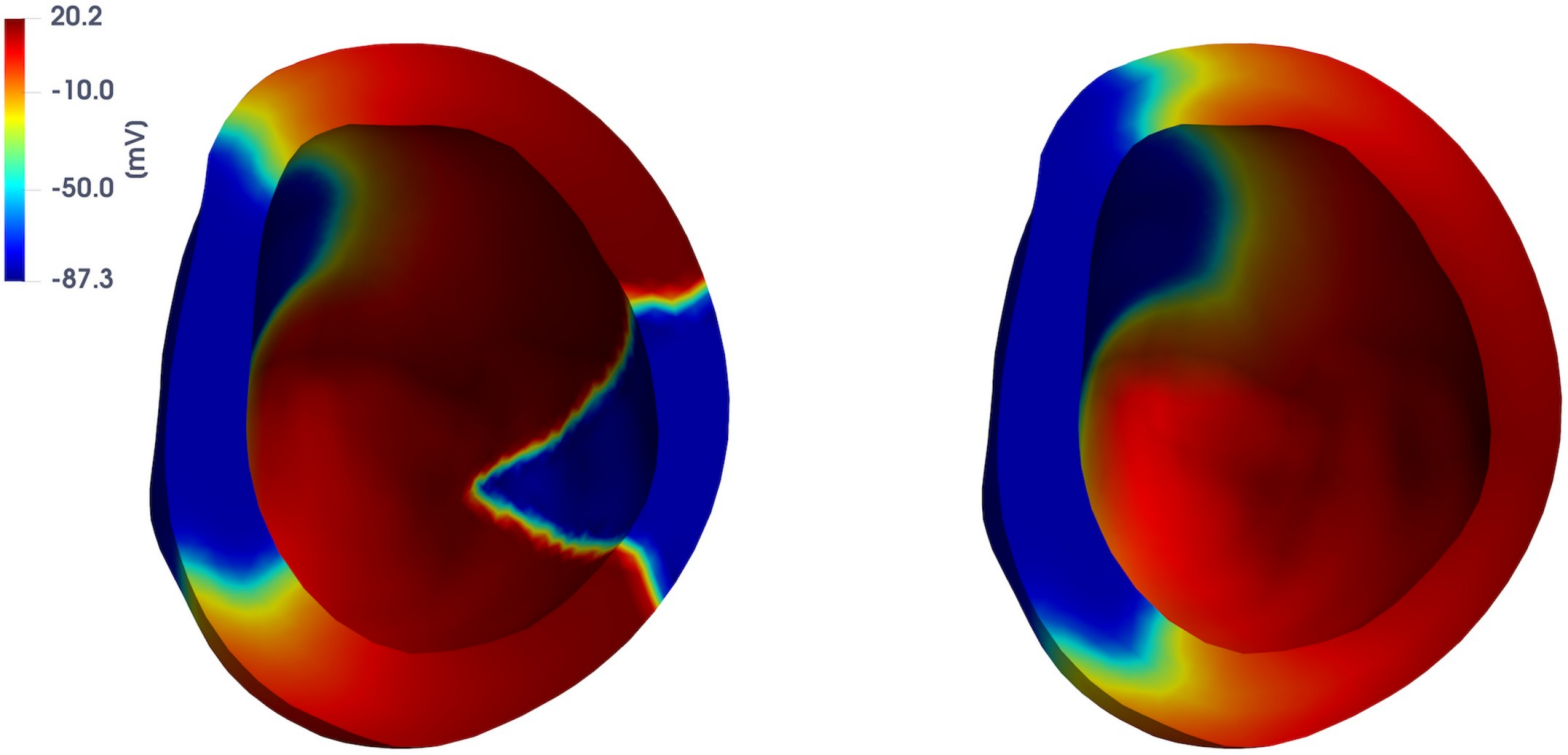
Test 3: FOM solutions for different testing-parameter instances. FOM solutions for *μ* = 12.9 ⋅ 0.0739 mm^2^/ms (left) and *μ* = 12.9 ⋅ 0.1991 mm^2^/ms (right) at $\tilde{t}=276$ ms. By increasing the value of *μ* the wavefront propagates faster.

In Fig 13 we report the FOM solution for two testing-parameter instances, i.e. *μ* = 12.9 ⋅ 0.0739 mm^2^/ms and *μ* = 12.9 ⋅ 0.1991 mm^2^/ms, at $\tilde{t}=276$ ms, to show the variability of the FOM solution over the parameter space. As expected, front propagation is faster for larger values of the parameter *μ*.

**Fig 14:**
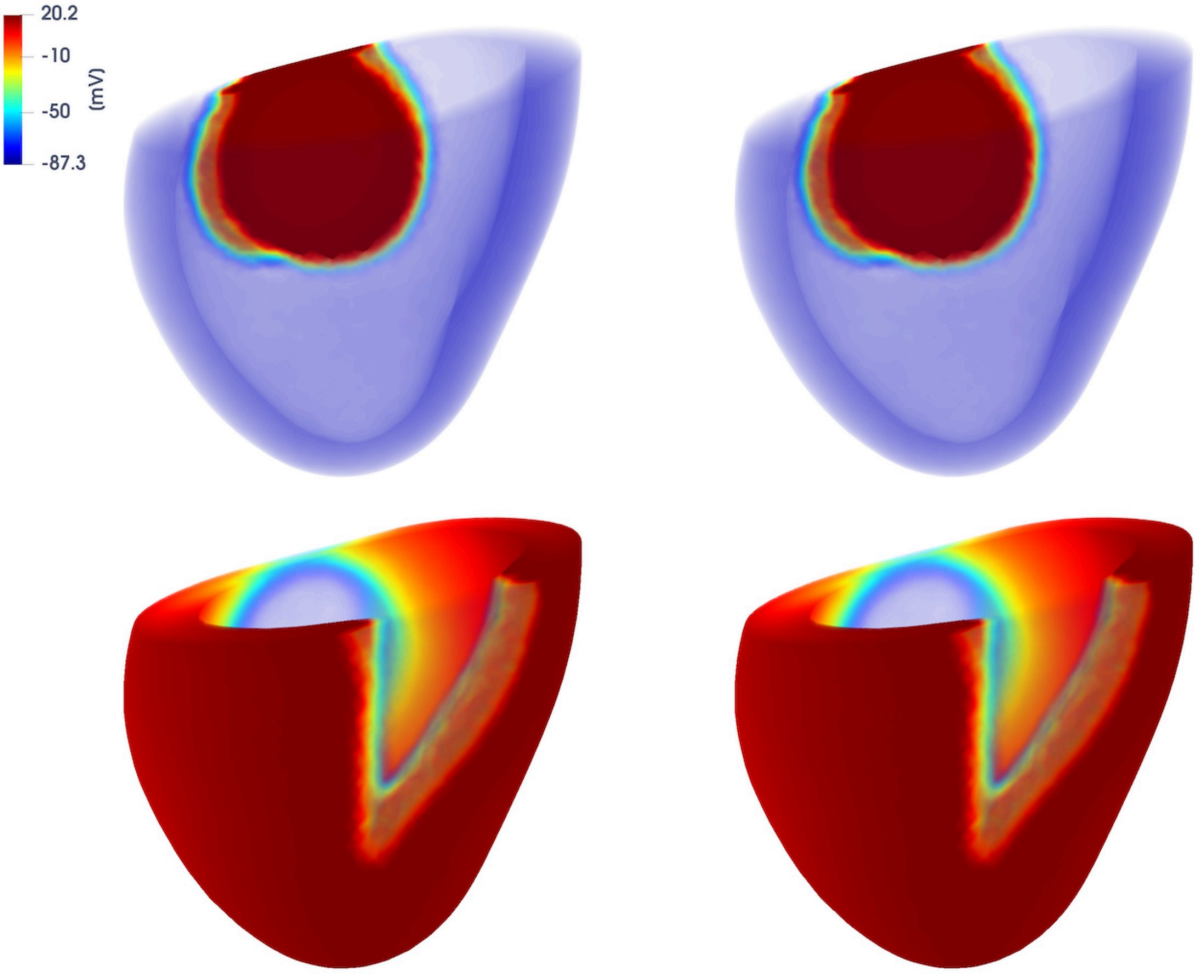
Test 3: Comparison between FOM and DL-ROM solutions for a testing-parameter instance at different time instances. FOM solution (left) and DL-ROM one (right), with *n* = 10, at $\tilde{t}=42.1$ ms (top) and $\tilde{t}=276$ ms (bottom), for the testing-parameter instance *μ*_*test*_ = 12.9 ⋅ 0.1435 mm^2^/ms.

**Fig 15:**
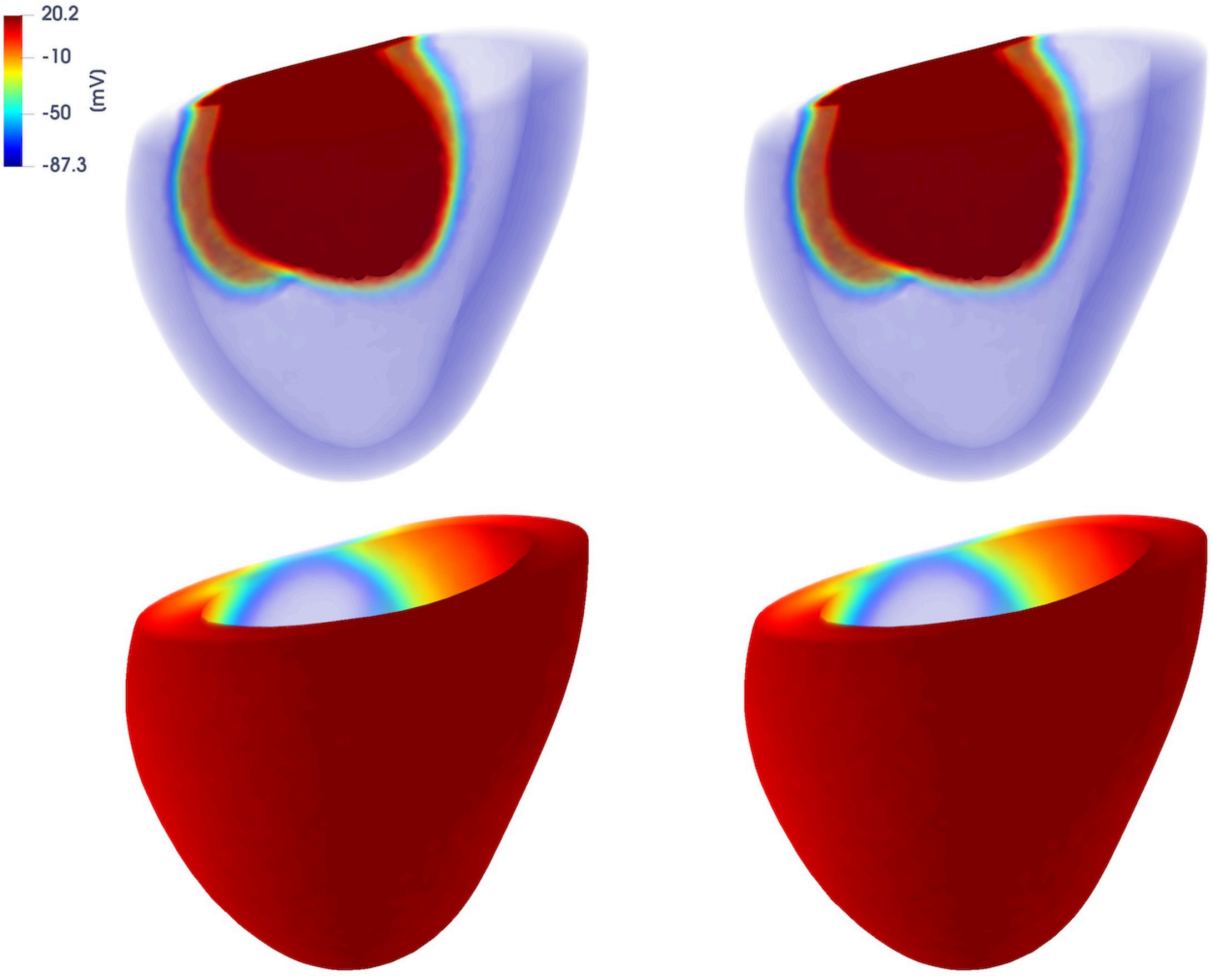
Test 3: Comparison between FOM and DL-ROM solutions for a testing-parameter instance at different time instances. FOM solution (left) and DL-ROM one (right), with *n* = 10, at $\tilde{t}=42.1$ ms (top) and $\tilde{t}=276$ ms (bottom), for the testing-parameter instance *μ*_*test*_ = 12.9 ⋅ 0.3243 mm^2^/ms.

In Figs 14 and 15 we report the FOM and DL-ROM solutions, the latter with *n* = 10, at $\tilde{t}=42.1$ ms and $\tilde{t}=222.1$ ms, for two testing-parameter instances, *μ*_*test*_ = 12.9 ⋅ 0.1435 mm^2^/ms and *μ*_*test*_ = 12.9 ⋅ 0.3243 mm^2^/ms. The DL-ROM approximation is essentially as accurate as the FOM solution.

Also for this test case, it is possible to build a reduced nonlinear trial manifold of dimension very close to the intrinsic one—*n*_*μ*_ + 1 = 2—as long as the maximum number of epochs *N*_*epochs*_ is increased; the choice *n* = 10 is obtained as the best trade-off between accuracy and efficiency of the DL-ROM approximation in this case.

**Fig 16:**
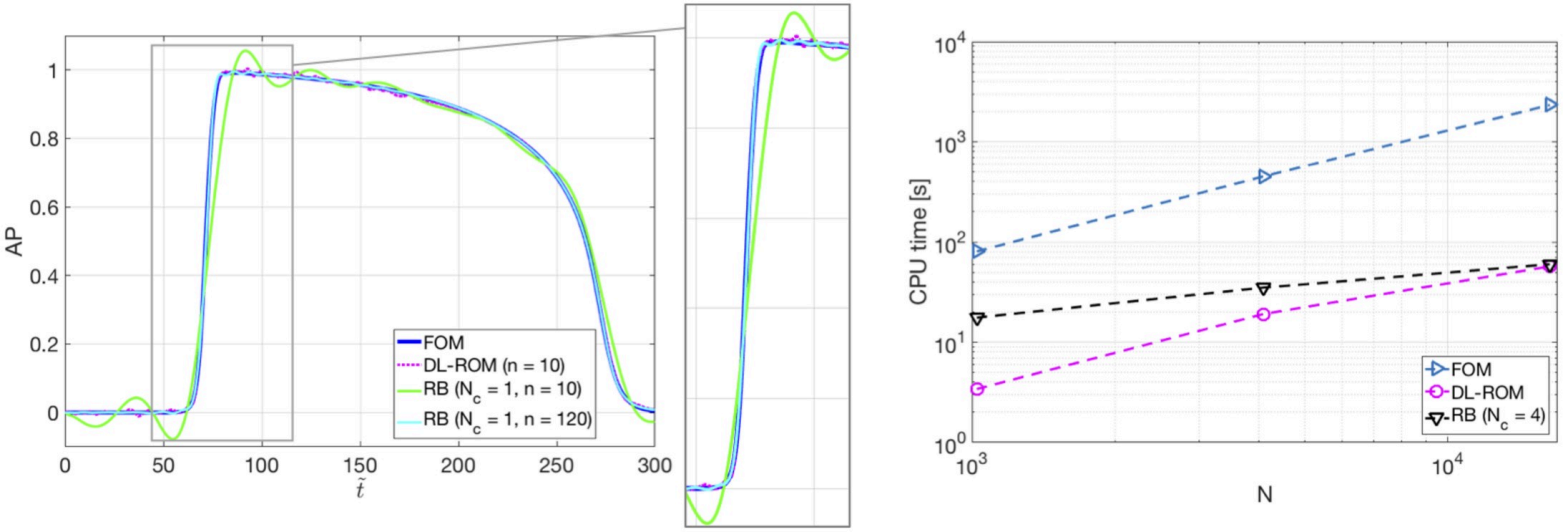
Test 3: FOM, DL-ROM and POD-Galerkin ROM APs for a testing-parameter instance. FOM and DL-ROM CPU computational times. Left: FOM, DL-ROM and POD-Galerkin ROM APs for the testing-parameter instance *μ*_*test*_ = 12.9 ⋅ 0.31 mm^2^/ms. For the same *n*, the DL-ROM is able to provide more accurate results than the POD-Galerkin ROM. Right: CPU time required to solve the FOM, by DL-ROM with *n* = 10 and by the POD-Galerkin ROM with *N*_*c*_ = 6 at testing time vs *N*. The DL-ROM is able to provide a speed-up equal to 41.

In Fig 16 (left) we report the APs obtained by the FOM and the DL-ROM, this latter with *n* = 10, computed at point *P* = [36.56; 1329.59; 28.82] mm for the testing-parameter instance *μ_test_* = 12.9 · 0.31 mm^2^/ms. For the sake of comparison, we also report the POD-Galerkin ROM approximation, with *N_c_* = 1, of dimension *n* = 10 and *n* = 120. Clearly, in dimension *n* = 10 the DL-ROM approximation is far more accurate than the POD-Galerkin ROM approximation; to reach the same accuracy (about *ϵ_rel_* = 5.9 × 10^−3^, measured through the error indicator (15)) achieved by the DL-ROM with *n* = 10, *n* = 120 POD modes would be required. In Fig 16 (right) we highlight instead the improvements, in terms of efficiency, enabled by the use of the DL-ROM technique; we report the CPU time required to solve the FOM for a testing-parameter instance, the one required by DL-ROM (of dimension n = 10) at testing time and by the POD-Galerkin ROM with *N_c_* = 4 (*n* = 68, 81, 82, 45), by using the time resolution each technique requires and by varying the FOM dimension *N* on a 6-core platform Numerical tests have been performed on a MacBook Pro Intel Core i7 6-core with 16 GB RAM. The FOM solution with *N* = 16365 degrees of freedom requires about 40 minutes to be computed, against 57 seconds required by the DL-ROM approximation, thus implying a speed-up almost equal to 41 times.

Regarding the training (offline) times for this test case, featuring a FOM dimension *N* = 16365, training the DL-ROM neural network on a GTX 1070 8GB GPU requires about 160 hours, whereas training the POD-Galerkin ROM (with *N*_*c*_ = 4 local bases) on a full node (20 Intel^®^Xeon^®^ E5-2640 v4 2.4GHz cores) of a HPC cluster requires about 28 hours; in both cases, the time needed to assemble the snapshot matrix **S** is not included. We report also the GPU testing computational time of DL-ROM which is equal to 0.35 seconds thus obtaining a speed-up, with respect the POD-Galerkin ROM testing time with *N*_*c*_ = 4, equal to 172. The efficiency introduced at testing time justifies the higher training time of DL-ROM.

### Test 4: Two-dimensional slab with varying restitution properties

To take into account a case in which parameters also affect the ionic model, we finally focus on the solution of problem (1) in a square slab of cardiac tissue Ω = (0, 10) cm, considering *n_**μ**_* = 3 parameters possibly reflecting intra- and inter- subjects variability. More precisely, the conductivity tensor now takes the form

$$D(x;\mu )=\mu_{2}I+(\mu_{1}-\mu_{2})f_{0}⊗f_{0},$$

where *μ*_1_ and *μ*_2_ consist of the electric conductivities in the longitudinal and the transversal direction to the fibers **f**_0_ = (1, 0)^*T*^, respectively, and *μ*_3_ regulates the action potential duration (APD) by defining

$$g(u,w)=(\epsilon_{0}+\frac{\mu_{3}w}{c_{2}+u})(-w-Ku(u-b-1)).$$

The parameters belong to the parameter space $P=12.9\cdot [0.06,0.2]{\text{cm}}^{2}/\text{ms}\times 12.9\cdot [0.03,0.1]{\text{cm}}^{2}/\text{ms}\times [0.15,0.25]$ and the applied current is defined as in Test 1.

For the training phase, we uniformly sample *N*_*t*_ = 1000 time instances in the interval (0, *T*) and consider *N*_*train*_ = 5 × 5 × 5 = 125 training-parameters, i.e. ***μ***_*train*_ = (12.9 ⋅ (0.06 + *i*0.035), 12.9 ⋅ (0.03 + *j*0.0175), 0.15 + *s*0.025) with *i*, *j*, *s* = 0, …, 4. For the testing phase, *N*_*test*_ = 16 testing-parameter instances have been considered, each of them given by ***μ***_*test*_ = (12.9 ⋅ (0.0775 + *i*0.035), 12.9 ⋅ (0.0387 + *j*0.0175), 0.1625+ *s*0.025) with *i*, *j*, *s* = 0, …, 3. The maximum number of epochs is *N*_*epochs*_ = 10000, the batch size is *N*_*b*_ = 40 and, regarding the early-stopping criterion, we stop the training if the loss function does not decrease along 500 epochs.

**Fig 17:**
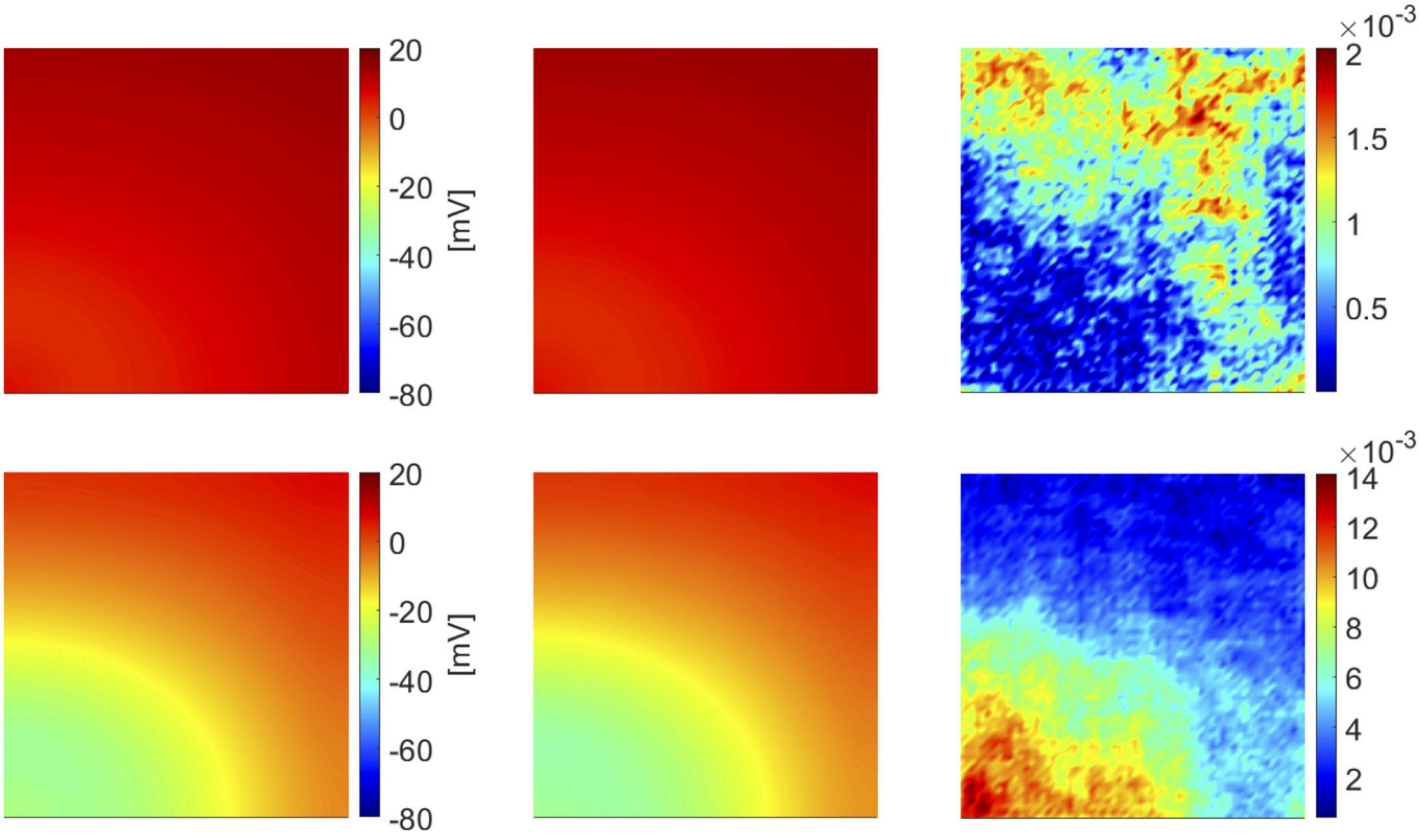
Test 4: Comparison between FOM and DL-ROM solutions for different testing-parameter instances. FOM solution (left), DL-ROM solution with *n* = 4 (center) and relative error ***ϵ***_*k*_ (right) for the testing-parameter instances ***μ**_text_* = (12.9 · 0.1125 cm^2^/ms, 12.9 · 0.0563 cm^2^/ms, 0.1875) (top) and ***μ**_text_* = (12.9 · 0.1475 cm^2^/ms, 12.9 · 0.0737 cm^2^/ms, 0.2375) (bottom) at $\tilde{t}=319.7$ ms. The maximum of the relative error ***ϵ***_*k*_ is about 10^−3^.

In Fig 17 we show the FOM and the DL-ROM approximation, this latter with *n* = 4, at $\tilde{t}=319.7$ ms for the testing-parameter innstaces ***μ**_test_* = (12.9 · 0.1125 cm^2^/ms, 12.9. 0.0563 cm^2^/ms, 0.1875) and ***μ**_test_* = (12.9 · 0.1475 cm^2^/ms, 12.9 · 0.0737 cm^2^/ms, 0.2375), together with the relative error defined as in (17). The DL-ROM technique is able to capture the strong variability of the solution over the parameter space. Indeed, in Fig 17 (top) the tissue is almost completely depolarized whereas in Fig 17 (bottom) repolarization has already started. The error indicator, computed as in (15) over these *N_test_* = 16 testing-parameter instances, is equal to 5.4 × 10^−3^.

**Fig 18:**
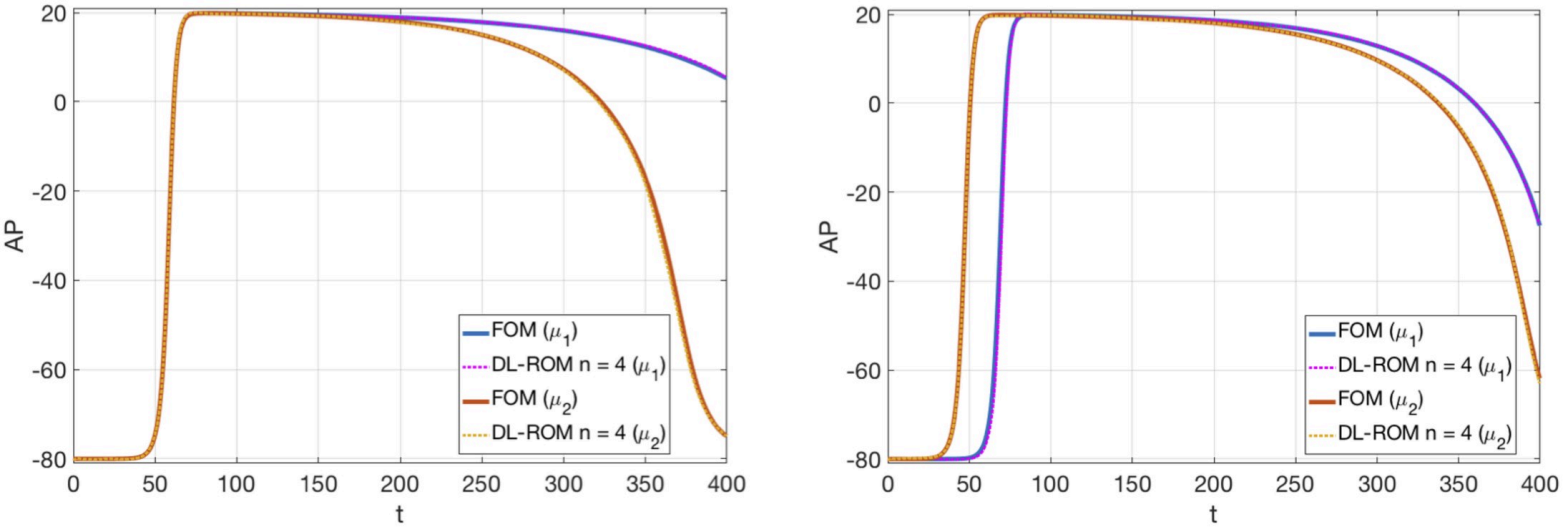
Test 4: Comparison between FOM and DL-ROM solutions for different testing-parameter instances. APs obtained through the FOM and the DL-ROM with *n* = 4. Left: $\mu_{test}^{1}=\left(12.9\cdot 0.1125\;{\text{cm}}^{2}/\text{ms},12.9\cdot 0.0737\;{\text{cm}}^{2}/\text{ms},0.1625\right)$ and $\mu_{test}^{2}=\left(12.9\cdot 0.1125\;{\text{cm}}^{2}/\text{ms},12.9\cdot 0.0737\;{\text{cm}}^{2}/\text{ms},0.2375\right)$; right: $\mu_{test}^{1}=\left(12.9\cdot 0.0775\;{\text{cm}}^{2}/\text{ms},12.9\cdot 0.0387\;{\text{cm}}^{2}/\text{ms},0.2125\right)$ and $\mu_{test}^{2}=\left(12.9\cdot 0.1825\;{\text{cm}}^{2}/\text{ms},12.9\cdot .0912\;{\text{cm}}^{2}/\text{ms},0.2125\right)$. The DL-ROM approximation accurately reproduces the APD variability and the different depolarization patterns.

In Fig 18 we compare the FOM and the DL-ROM APs at **x** = (9.524, 4.762) cm, by considering the effect of the different parameters separately. More precisely, in Fig 18 (left) we let *μ*_3_ vary, i.e. we take $\mu_{test}^{1}=(12.9\cdot 0.1125\;{\text{cm}}^{2}/\text{ms},12.9\cdot 0.0737\;{\text{cm}}^{2}/\text{ms},0.1625)$ and $\mu_{test}^{2}=(12.9\cdot 0.1125\;{\text{cm}}^{2}/\text{ms},12.9\cdot 0.0737\;{\text{cm}}^{2}/\text{ms},0.2375)$. In Fig 18 (right) instead we only vary *μ*_1_ and *μ*_2_, i.e. we take $\mu_{test}^{1}=(12.9\cdot 0.0775{\text{ cm}}^{2}/\text{ms},12.9\cdot 0.0387{\text{ cm}}^{2}/\text{ms},0.2125)$ and $\mu_{test}^{2}=(12.9\cdot 0.01825{\text{ cm}}^{2}/\text{ms},12.9\cdot 0.0912{\text{ cm}}^{2}/\text{ms},0.2125)$. In both cases, the DL-ROM correctly reproduces the APD variability and the different depolarization patterns.

Finally, we report in Table 4 the training and testing computational times of the DL-ROM, on a GTX 1070 8 GB GPU, by considering either *n_μ_* = 2 or 3 parameters:

- *n_μ_* = 2, *N_rain_* = 5 × 5 = 25, *N_t_* = 1000, with $P=12.9\cdot [0.06,0.2]{\text{cm}}^{2}/\text{ms}\times 12.9\cdot [0.03,0.1]{\text{cm}}^{2}/\text{ms},$
- *n_μ_* = 3, *N_rain_* = 5 × 5 × 5 = 125, *N_t_* = 1000, with $P=12.9\cdot [0.06,0.2]{\text{cm}}^{2}/\text{ms}\times 12.9\cdot [0.03,0.1]{\text{cm}}^{2}/\text{ms}\times [0.15,0.25],$

to analyze the effect of introducing an additional parameter on the training time for a prescribed level of accuracy, keeping the architecture of the network fixed. The training time refers to the overall training and validation time, while the testing one refers to the time needed by the DL-ROM to compute *N_t_* time instances of the solution for a given testing-parameter instance. In this case, considering *N_train_* = 125 training-parameter instances allows us to reduce the training computational time of a factor 3, even if more parameters are considered, a larger training set is provided. However, we highlight that stating general conclusions about the training complexity and costs, as a function of the number of parameters and the training set dimensions, is far from being trivial, and still represents an open issue in this framework.

**Table 4:** Test 4: DL-ROM training and testing computational times.

*n*_*μ*_	*N*_*train*_	train. time	*n*_*epochs*_	test.time
2	25	15 h	6981	0.08 s
3	125	5 h	449	0.08 s

Number of parameters, of training-parameter instances and of epochs together with training and testing computational times in the two configurations.

## Conclusion

In this work we have proposed a new efficient reduced order model obtained using deep learning algorithms to boost the solution of parametrized problems in cardiac electrophysiology. Numerical results show that the resulting DL-ROM technique, formerly introduced in [26], allows one to accurately capture complex wave propagation processes, both in physiological and pathological scenarios.

The proposed DL-ROM technique provides ROMs that are orders of magnitude more efficient than the ones provided by common linear (projection-based) ROMs, built for instance through a POD-Galerkin reduced basis method, for a prescribed level of accuracy. Through the use of DL-ROM, it is possible to overcome the main computational bottlenecks shown by POD-Galerkin ROMs, when addressing parametrized problems in cardiac electrophysiology. The most critical points related to (i) the linear superimposition of modes which linear ROMs are based on; (ii) the need to account for the gating variables when solving the reduced dynamics, even if not required; and (iii) the necessity to use (very often, expensive) hyper-reduction techniques to deal with terms that depend nonlinearly on either the transmembrane potential or the input parameters, are all addressed by the DL-ROM technique, which finally yields more efficient and accurate approximation than POD-Galerkin ROMs. Moreover, larger time resolutions can be employed when using a DL-ROM, compared to the ones required by the numerical solution of a dynamical systems through a FOM or a POD-Galerkin ROM. Indeed, the DL-ROM approximation can be queried at any desired time, without requiring to solve a dynamical system until that time, thus drastically decreasing the computational time required to compute the approximated solution at any given time.

Outputs of clinical interest, such as activation maps and action potentials, can be more efficiently evaluated by the DL-ROM technique than by a FOM built through the finite element method, while maintaining a high level of accuracy. This work is a proof-of-concept of the DL-ROM technique ability to investigate intra- and inter- subjects variability, towards performing multi-scenario analyses in real time and, ultimately, supporting decisions in clinical practice. In this respect, the use of DL-ROM techniques can foster assimilation of clinical data with physics-driven computational models.

So far, the training time required by the DL-ROM technique appears to be the major computational bottleneck, even if it is completely compensated by the great computational efficiency provided at testing time. Enhancing efficiency also during the training phase represents the focus of our ongoing research activity, and will be the object of a forthcoming publication.

## Supporting information

S1 Appendix Derivation of the FOM. We provide the complete derivation of the spatial (and temporal) discretization of system (1).
(PDF)

S2 Appendix DL-ROM neural network architecture. Here we report the configuration of the DL-ROM neural network used for our numerical tests.
(PDF)

S1 File Features maps. In the notes we report the feature maps of the DL-ROM neural network.
(PDF)

S2 File Fibers field distribution. Here we report the resulting fibers distribution used for Test 3.
(PDF)

S3 File Activation maps. Here we show the ability of the DL-ROM approximation to replace the FOM solution when evaluating outputs of interest.
(PDF)

S1 Code Code and data. (TXT)

S1 Fig (EPS)

S2 Fig (EPS)

S3 Fig (EPS)
