# Supplementary material for: Deep learning-based reduced order models in cardiac electrophysiology
Source: PLoS One. 2020 Oct 1;15(10):e0239416. doi: 10.1371/journal.pone.0239416 (PMC7529269; doi:10.1371/journal.pone.0239416)
Supplement: S1 Appendix — We provide the complete derivation of the spatial (and temporal) discretization of system (1). (PDF) [file pone.0239416.s001.pdf]

Deep learning-based reduced order models in cardiac electrophysiology

Stefania Fresca[1*], Andrea Manzoni[1], Luca Dedé[1], Alfio Quarteroni[1,2]

1 MOX - Dipartimento di Matematica, Politecnico di Milano, Milano, Italy
2 Mathematics Institute, École Polytechnique Fédérale de Lausanne, Lausanne, Switzerland

* stefania.fresca@polimi.it

S1 Appendix.

To make the paper self-contained, in this section we provide the complete derivation of the spatial (and temporal) discretization of system (1), constituting our full-order model (FOM). Hereon we denote by $\boldsymbol{\mu} \in \mathcal{P} \subset \mathbb{R}^{n_{\boldsymbol{\mu}}}$ a parameter vector listing the $n_{\boldsymbol{\mu}}$ input parameters of interest characterizing physical (and, possibly, geometrical) properties; moreover, whenever clear we omit the dependence on the spatial.variables $\mathbf{x}$.

We first state the weak formulation of problem (1), which stands at the basis of the full-order approximation of the problem, obtained with the Galerkin-finite element (FE) method. For $t > 0$, the weak formulation of problem (1) reads: given $I_{app}(t;\boldsymbol{\mu}) \in L^2(\Omega(\boldsymbol{\mu}))$, find $u(t;\boldsymbol{\mu}) \in X = H^1(\Omega(\boldsymbol{\mu}))$ and $w(t;\boldsymbol{\mu}) \in L^2(\Omega(\boldsymbol{\mu}))$ such that

$$\begin{aligned}
\int_{\Omega(\boldsymbol{\mu})} \left(\frac{\partial u}{\partial t} + I_{ion}(u, w; \boldsymbol{\mu}) \right) \psi d\mathbf{x} + \int_{\Omega(\boldsymbol{\mu})} \mathbf{D}(\boldsymbol{\mu}) \nabla v \cdot \nabla \psi d\mathbf{x} = \int_{\Omega(\boldsymbol{\mu})} I_{app}(t;\boldsymbol{\mu}) \psi d\mathbf{x} \quad \forall \psi \in H^1(\Omega(\boldsymbol{\mu})),\\
\int_{\Omega(\boldsymbol{\mu})} \frac{\partial w}{\partial t} \eta d\mathbf{x} = \int_{\Omega(\boldsymbol{\mu})} g(u, w; \boldsymbol{\mu}) \eta d\mathbf{x} \quad \forall \eta \in L^2(\Omega(\boldsymbol{\mu})),\\
u(0;\boldsymbol{\mu}) = 0, \quad w(0;\boldsymbol{\mu}) = 0,
\end{aligned}$$

where $\Omega(\boldsymbol{\mu})$ is a Lipschitz domain of $\mathbb{R}^p$, $p = 2, 3$, possibly depending on a set of geometrical parameters. In this paper, we focus on the Aliev-Panfilov model, which consists in taking

$$\begin{aligned}
I_{ion}(u, w) &= Ku(u-a)(u-1) + uw,\\
g(u, w) &= \left(\epsilon_0 + \frac{c_1 w}{c_2 + u} \right)(-w - Ku(u - b - 1)).
\end{aligned}$$

We then apply the Galerkin-FE method on a finite-dimensional space $X_h \subset X(\Omega)$ of (usually very large) dimension $\dim(X_h) = N$; here by h we denote a parameter related to the mesh size of the computational grid. By denoting with $\{\varphi_i\}_{j=1}^N$ a set of basis functions of the FE space X_h, we express the discrete approximation to $u(\mathbf{x}, t; \boldsymbol{\mu})$ and $w(\mathbf{x}, t; \boldsymbol{\mu})$ by

$$u_h(\mathbf{x}, t; \boldsymbol{\mu}) = \sum_{i=1}^N u_i(t;\boldsymbol{\mu}) \varphi_i(\mathbf{x}), \qquad w_h(\mathbf{x}, t; \boldsymbol{\mu}) = \sum_{i=1}^N w_i(t;\boldsymbol{\mu}) \varphi_i(\mathbf{x}),$$

where the vectors $\mathbf{u} = [u_1, \dots, u_N]^T$ and $\mathbf{w} = [w_1, \dots, w_N]^T$ are obtained by solving the following discrete system: given $\boldsymbol{\mu} \in \mathcal{P}$, find $\mathbf{u} = \mathbf{u}(t;\boldsymbol{\mu})$ and $\mathbf{w} = \mathbf{w}(t;\boldsymbol{\mu})$ such that

$$\begin{cases}
\mathbf{M}(\boldsymbol{\mu}) \dfrac{\partial \mathbf{u}}{\partial t} + \mathbf{A}(\boldsymbol{\mu})\mathbf{u} + \mathbf{I}_{ion}(t, \mathbf{u}, \mathbf{w}; \boldsymbol{\mu}) = \mathbf{I}_{app}(t;\boldsymbol{\mu}), & t \in (0, T)\\
\dfrac{\partial \mathbf{w}_h}{\partial t} = g(\mathbf{u}, \mathbf{w}), & t \in (0, T)\\
\mathbf{u}(0;\boldsymbol{\mu}) = \mathbf{u}_0(\boldsymbol{\mu}), \quad \mathbf{w}(0;\boldsymbol{\mu}) = \mathbf{w}_0(\boldsymbol{\mu}).
\end{cases} \tag{1}$$

which is nothing but (the $\boldsymbol{\mu}$-dependent version of) equation (4). Here we denote the $\boldsymbol{\mu}$-dependent mass matrix, the stiffness matrix and the activation term by

$$(\mathbf{M}(\boldsymbol{\mu}))_{ij} = \int_{\Omega(\boldsymbol{\mu})} \varphi_i \varphi_j d\mathbf{x}, \quad (\mathbf{A}(\boldsymbol{\mu}))_{ij} = \int_{\Omega(\boldsymbol{\mu})} \mathbf{D}(\boldsymbol{\mu}) \nabla \varphi_i \cdot \nabla \varphi_j d\mathbf{x}, \quad (\mathbf{I}_{app}(t;\boldsymbol{\mu}))_j = \int_{\Omega(\boldsymbol{\mu})} I_{app}(t;\boldsymbol{\mu}) \varphi_j d\mathbf{x},$$

respectively; the $\boldsymbol{\mu}$-dependent vectors accounting for the ionic terms are instead given by

$$(\mathbf{I}_{ion}(\mathbf{u}_h, \mathbf{w}_h; \boldsymbol{\mu}))_j = \int_{\Omega(\boldsymbol{\mu})} I_{ion}(\mathbf{u}_h, \mathbf{w}_h; \boldsymbol{\mu}) \varphi_j d\mathbf{x}, \quad (\mathbf{g}(\mathbf{u}_h, \mathbf{w}_h; \boldsymbol{\mu}))_j = \int_{\Omega(\boldsymbol{\mu})} g(\mathbf{u}_h, \mathbf{w}_h; \boldsymbol{\mu}) \varphi_j d\mathbf{x}.$$

Concerning the treatment of nonlinear terms and time discretization, we rely a semi-implicit, first order, one-step scheme [1]. Given a partition (t^k, t^{k+1}), $k = 0, \ldots, N_t - 1$ of $(0, T)$ into N_t subintervals of length Δt, at each time-step t^{k+1} the nonlinear vector $\mathbf{I}_{ion}$ is evaluated around the solution already computed at time t^k. In this way, we can decouple the PDE from the ODE, thus obtaining a linear system to be solved at each time step. Moreover, a ionic current interpolation strategy is used to evaluate the ionic current term, so that only the nodal values are used to build a (piecewise linear) interpolant of the ionic current. This is one of the two most common ways to deal with the evaluation of the ionic current at the quadrature nodes, and ultimately with the numerical integration of the ionic term I_{ion}, which go under the name of *state variable interpolation* (SVI) and *ionic current interpolation* (ICI), see, e.g., [2,3]:

- When using SVI, the variables v_h, w_h are evaluated at the quadrature nodes $\bar{\mathbf{x}}_q$, $q = 1, \ldots, N_Q$, so that

$$\int_{\Omega(\boldsymbol{\mu})} I_{ion}(\mathbf{u}_h, \mathbf{w}_h; \boldsymbol{\mu}) \varphi_j d\mathbf{x} \approx \sum_{q=1}^{N_Q} I_{ion} \left(\sum_{i=1}^{N} u_i(\boldsymbol{\mu}) \varphi_i(\mathbf{x}_q), \sum_{i=1}^{N} w_i(\boldsymbol{\mu}) \varphi_i(\mathbf{x}_q) \right) \boldsymbol{\omega}_q$$

  where $\{\boldsymbol{\omega}_q\}_{q=1}^{N_Q}$ denote the corresponding quadrature weights. This approach corresponds to the standard Galerkin-FEM method.

- When relying on ICI [2], the currents are first evaluated in the degrees of freedom, and then interpolated at the quadrature nodes, so that

$$\int_{\Omega(\boldsymbol{\mu})} I_{ion}(\mathbf{u}_h, \mathbf{w}_h; \boldsymbol{\mu}) \varphi_j d\mathbf{x} \approx \sum_{q=1}^{N_Q} \sum_{i=1}^{N} I_{ion} \left(u_i(\boldsymbol{\mu}), w_i(\boldsymbol{\mu}) \right) \varphi_i(\mathbf{x}_q) \boldsymbol{\omega}_q,$$

  in order to reduce the computational cost associated to the assembly of the ionic currents term, compared to the SVI case.

Moreover, we also remark that since the ionic currents (zero order) term dominates the diffusion (second order) term, a known numerical issue might occur, ultimately causing numerical instabilities; to avoid them, we replace the mass matrix with a lumped mass matrix.

In conclusion, the fully discrete version of the full-order model (FOM) reads as: given $\boldsymbol{\mu} \in \mathcal{P}$, find $\mathbf{u}^{k+1} = \mathbf{u}^{k+1}(\boldsymbol{\mu})$ and $\mathbf{w}^{k+1} = \mathbf{w}^{k+1}(\boldsymbol{\mu})$ such that $\mathbf{u}^{(0)} = \mathbf{0}$, $\mathbf{w}^{(0)} = \mathbf{0}$ and, for $k = 0, \ldots, N_t - 1$,

$$\begin{cases} \dfrac{\mathbf{w}^{k+1} - \mathbf{w}^k}{\Delta t} - \mathbf{g}(\mathbf{u}^k, \mathbf{w}^{k+1}; \boldsymbol{\mu}) = \mathbf{0}, \\ \mathbf{M}(\boldsymbol{\mu}) \dfrac{\mathbf{u}^{k+1} - \mathbf{u}^k}{\Delta t} + \mathbf{A}(\boldsymbol{\mu}) \mathbf{u}^{k+1} + \mathbf{I}_{ion}(\mathbf{u}^k, \mathbf{w}^{k+1}; \boldsymbol{\mu}) - \mathbf{I}_{app}^{k+1}(\boldsymbol{\mu}) = \mathbf{0}. \end{cases} \tag{2}$$

References

1. Colli Franzone P, Pavarino LF. A parallel solver for reaction–diffusion systems in computational electrocardiology. Mathematical Models and Methods in Applied Sciences. 2004;14(06):883–911.

2. Pathmanathan P, Chapman S, Gavaghan D, Whiteley J. Cardiac electromechanics: the effect of contraction model on the mathematical problem and accuracy of the numerical scheme. The Quarterly Journal of Mechanics and Applied Mathematics. 2010;27(11):1751–1770.

3. Pathmanathan P, Bernabeu M, Niederer S, Gavaghan D, Kay D. Computational modelling of cardiac electrophysiology: ex- planation of the variability of results from different numerical solvers. International Journal for Numerical Methods in Biomedical Engineering. 2012;28(8):890–903.
