# Supplementary material for: Deep learning-based reduced order models in cardiac electrophysiology
Source: PLoS One. 2020 Oct 1;15(10):e0239416. doi: 10.1371/journal.pone.0239416 (PMC7529269; doi:10.1371/journal.pone.0239416)
Supplement: S2 Appendix — Here we report the configuration of the DL-ROM neural network used for our numerical tests. (PDF) [file pone.0239416.s002.pdf]

Deep learning-based reduced order models in cardiac electrophysiology

Stefania Fresca[1*], Andrea Manzoni[1], Luca Dedé[1], Alfio Quarteroni[1,2]

1 MOX - Dipartimento di Matematica, Politecnico di Milano, Milano, Italy
2 Mathematics Institute, École Polytechnique Fédérale de Lausanne, Lausanne, Switzerland

* stefania.fresca@polimi.it

S2 Appendix.

DL-ROM neural network architecture. Here we report the configuration of the DL-ROM neural network used for our numerical tests. We employ a 12-layers DFNN equipped with 50 neurons per hidden layer and n neurons in the output layer, where n corresponds to the dimension of the reduced nonlinear trial manifold. The architectures of the encoder and decoder functions are instead reported in Table 1 and 2.

Layer	Input dimension	Output dimension	Kernel size	# of filters	Stride	Padding
1			[5, 5]	8	1	SAME
2			[5, 5]	16	2	SAME
3			[5, 5]	32	2	SAME
4			[5, 5]	64	2	SAME
5	N	256				
6	256	n				

Table 1. Attributes of convolutional and dense layers in the encoder $\mathbf{f}_n^E$.

Layer	Input dimension	Output dimension	Kernel size	# of filters	Stride	Padding
1	n	256				
2	256	N				
3			[5, 5]	64	2	SAME
4			[5, 5]	32	2	SAME
5			[5, 5]	16	2	SAME
6			[5, 5]	1	1	SAME

Table 2. Attributes of dense and transposed convolutional layers in the decoder $\mathbf{f}^D$.
