# Supplementary material for: Deep learning-based reduced order models in cardiac electrophysiology
Source: PLoS One. 2020 Oct 1;15(10):e0239416. doi: 10.1371/journal.pone.0239416 (PMC7529269; doi:10.1371/journal.pone.0239416)
Supplement: S1 File — In the notes we report the feature maps of the DL-ROM neural network. (PDF) [file pone.0239416.s003.pdf]

Deep learning-based reduced order models in cardiac electrophysiology

Stefania Fresca[1*], Andrea Manzoni[1], Luca Dedé[1], Alfio Quarteroni[1,2]

1 MOX - Dipartimento di Matematica, Politecnico di Milano, Milano, Italy
2 Mathematics Institute, École Polytechnique Fédérale de Lausanne, Lausanne, Switzerland

[*] stefania.fresca@polimi.it

S1 File.

Features maps. Here we report the feature maps of the DL-ROM neural network. In Figs 1 we show the feature maps of the first convolutional layer of the encoder function $\sigma_1(W_1^k * \mathbf{u}^1(\boldsymbol{\mu_{test}}) + b_1^k)$, for $k = 1, \ldots, 8$, in the DL-ROM neural network when the FOM solution for the testing-parameter instances $\boldsymbol{\mu}_{test} = (3.75, 3.75)$ cm and $\boldsymbol{\mu}_{test} = (6.25, 6.25)$ cm at $t = 0.2$ ms, are provided as inputs. At this stage, the feature maps retain most of the information present in the FOM solution. Moreover, by considering the two testing-parameter instances, we observe the translation equi-variance property [1] that convolutional layers hold when applied to the part of cardiac tissue corresponding to the scar. Moving to deeper layers, feature maps become increasingly abstract, and less visually interpretable; however, the extracted high-level features are still related both to the ischemic region and the electrical activation pattern.

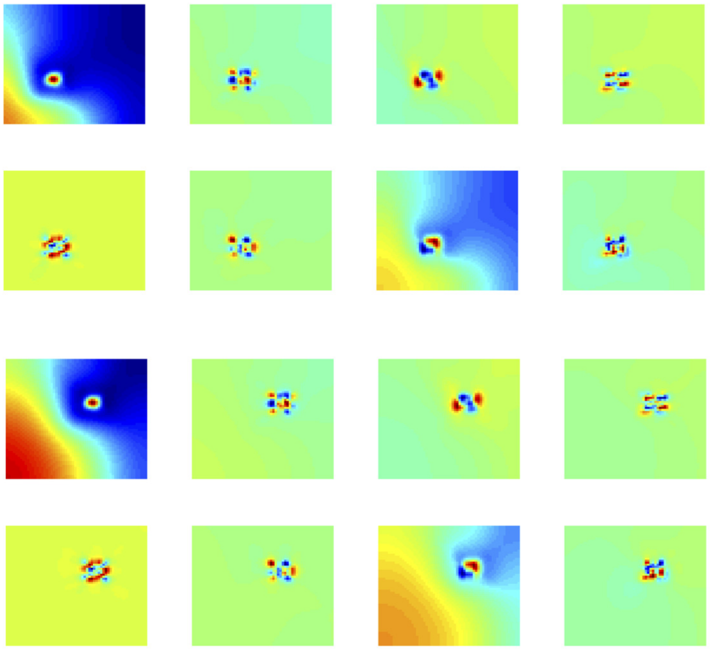

Fig 1. Test 1: activations of the first convolutional layer of the encoder function for a testing-parameter instance. Feature maps of the first convolutional layer of the encoder function in the DL-ROM neural network for the testing-parameter instances $\boldsymbol{\mu}_{test} = (3.75, 3.75)$ cm (top) and $\boldsymbol{\mu}_{test} = (6.25, 6.25)$ cm (bottom) at $\tilde{t} = 0.2$ ms.

References

1. Goodfellow I, Bengio Y, Courville A. Deep Learning. MIT Press; 2016. Available from: http://www.deeplearningbook.org.
