# Supplementary material for: Deep learning-based reduced order models in cardiac electrophysiology
Source: PLoS One. 2020 Oct 1;15(10):e0239416. doi: 10.1371/journal.pone.0239416 (PMC7529269; doi:10.1371/journal.pone.0239416)
Supplement: S2 File — Here we report the resulting fibers distribution used for Test 3. (PDF) [file pone.0239416.s004.pdf]

Deep learning-based reduced order models in cardiac electrophysiology

Stefania Fresca[1*], Andrea Manzoni[1], Luca Dedé[1], Alfio Quarteroni[1,2]

1 MOX - Dipartimento di Matematica, Politecnico di Milano, Milano, Italy
2 Mathematics Institute, École Polytechnique Fédérale de Lausanne, Lausanne, Switzerland

* stefania.fresca@polimi.it

S2 File.

Fibers field distribution. Here we show the resulting fibers distribution used for Test 3.

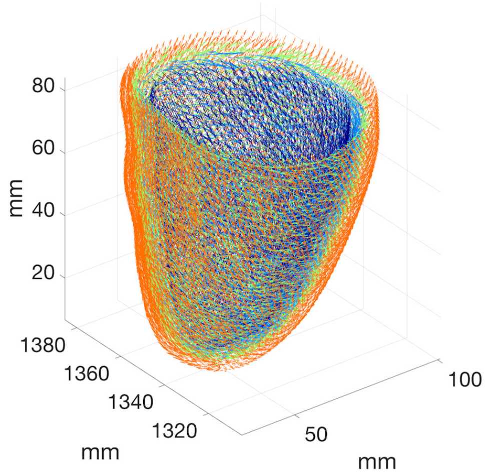

Fig 1. Test 3: fibers distribution. Fibers field on the Zygote LV geometry.
