# Supplementary material for: Deep learning-based reduced order models in cardiac electrophysiology
Source: PLoS One. 2020 Oct 1;15(10):e0239416. doi: 10.1371/journal.pone.0239416 (PMC7529269; doi:10.1371/journal.pone.0239416)
Supplement: S3 File — Here we show the ability of the DL-ROM approximation to replace the FOM solution when evaluating outputs of interest. (PDF) [file pone.0239416.s005.pdf]

Deep learning-based reduced order models in cardiac electrophysiology

Stefania Fresca[1*], Andrea Manzoni[1], Luca Dedé[1], Alfio Quarteroni[1,2]

1 MOX - Dipartimento di Matematica, Politecnico di Milano, Milano, Italy
2 Mathematics Institute, École Polytechnique Fédérale de Lausanne, Lausanne, Switzerland

* stefania.fresca@polimi.it

S3 File.

Activation maps. Here we show the ability of the DL-ROM approximation to replace the FOM solution when evaluating outputs of interest. For instance, in Fig 1 we show the FOM and DL-ROM activation maps, the latter obtained by choosing $n = 10$ as DL-ROM dimension. Given the electric potential $u = u(\mathbf{x}, t; \boldsymbol{\mu})$, the (unipolar) activation map at a point $\mathbf{x} \in \Omega$ is evaluated as the minimum time at which the AP peak reaches $\mathbf{x}$, that is,

$$AC(\mathbf{x}; \boldsymbol{\mu}) = \arg \min_{t \in (0,T)} \left(u(\mathbf{x}, t; \boldsymbol{\mu}) = \max_{t \in (0,T)} u(\mathbf{x}, t; \boldsymbol{\mu}) \right).$$

Here we compare the activation maps AC_{FOM} and AC_{DL-ROM} obtained through the FOM and the DL-ROM, respectively, by evaluating the maximum of the relative error

$$\boldsymbol{\epsilon}_{AC}(\mathbf{x}; \boldsymbol{\mu}) = \frac{|AC_{FOM}(\mathbf{x}; \boldsymbol{\mu}) - AC_{DL-ROM}(\mathbf{x}; \boldsymbol{\mu})|}{|AC_{FOM}(\mathbf{x}; \boldsymbol{\mu})|}$$

over the N mesh points; in the case $\mu_{test} = 12.9 \cdot 0.31$, the maximum relative error is equal to 4.32×10^{-5}.

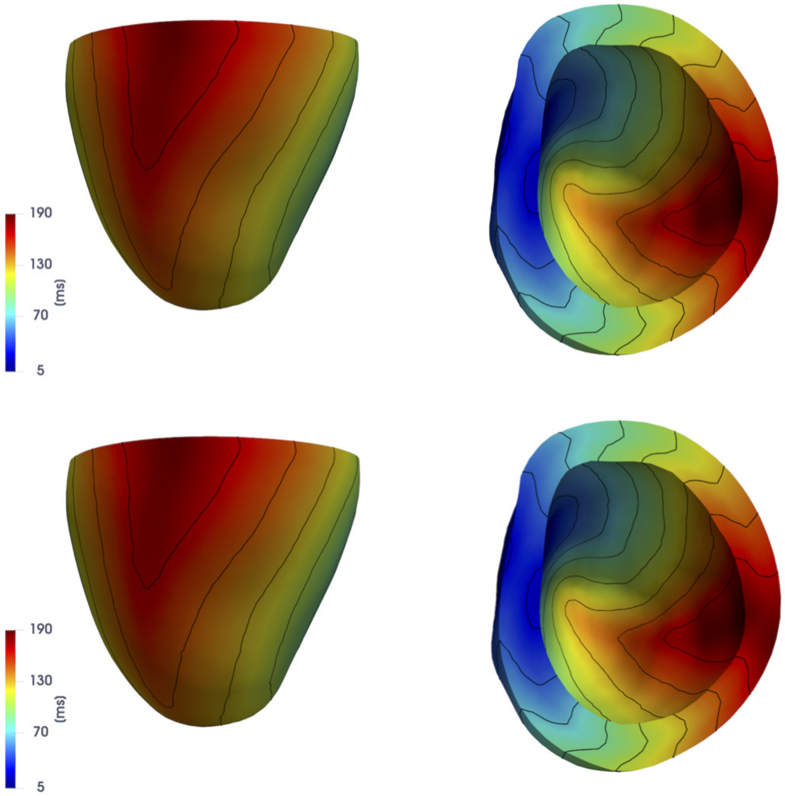

Fig 1. Test 3. FOM and DL-ROM activation maps. FOM (top) and DL-ROM (bottom) activation maps for the testing-parameter instance $\mu_{test} = 12.9 \cdot 0.31$ mm^2/ms with $n = 10$.
